# Development of CAR NK Cell Lines Selectively Targeting Cancer Cells Expressing Membrane Hsp70

**DOI:** 10.1002/mco2.70753

**Published:** 2026-05-10

**Authors:** Khouloud Hachani, Mina Yazdi, Charlotte Carcopino, Fatemeh Khademi Moghadam, Micholas Dean Smith, Anskar Trill, Marcel P. Trefny, Morteza Hasanzadeh Kafshgari, Cosima C. Hoch, Abdallah Gaballa, Bayan Alkotub, Jennifer Altomonte, A. Graham Pockley, Ernst Wagner, Barbara Wollenberg, Sebastian Kobold, Gabriele Multhoff, Ali Bashiri Dezfouli

**Affiliations:** ^1^ Department of Otolaryngology Head and Neck Surgery TUM School of Medicine and Health Technical University of Munich Munich Germany; ^2^ Department of Pharmacy Pharmaceutical Biotechnology Ludwig‐Maximilians‐Universität (LMU) Munich Germany; ^3^ Division of Clinical Pharmacology Department of Medicine IV University Hospital Ludwig‐Maximilians‐Universität (LMU) Munich Germany; ^4^ Department of Biochemistry and Cellular and Molecular Biology The University of Tennessee Knoxville Tennessee USA; ^5^ Central Institute for Translational Cancer Research (TranslaTUM) TUM School of Medicine and Health Technical University of Munich Munich Germany; ^6^ Heinz‐Nixdorf‐Chair of Biomedical Electronics Campus Klinikum München rechts der Isar TranslaTUM Technical University of Munich Munich Germany; ^7^ Division of Translational Cancer Research German Cancer Research Center and German Cancer Consortium Heidelberg Germany; ^8^ Chair of Translational Cancer Research and Institute of Experimental Cancer Therapy Klinikum Rechts der Isar School of Medicine Technical University of Munich Munich Germany; ^9^ Center for Translational Cancer Research (TranslaTUM) School of Medicine Technical University of Munich Munich Germany; ^10^ Institute of Biological and Medical Imaging Bioengineering Center Helmholtz Zentrum München Neuherberg Germany; ^11^ Chair of Biological Imaging Central Institute for Translational Cancer Research (TranslaTUM) School of Medicine and Health Technical University of Munich Munich Germany; ^12^ Department of Internal Medicine II Klinikum Rechts der Isar Technical University of Munich Munich Germany; ^13^ multimmune GmbH Munich Germany; ^14^ German Cancer Consortium (DKTK) a Partnership Between LMU University Hospital and DKFZ Heidelberg Germany; ^15^ German Center for Lung Research (DZL) Partner Site Munich Munich Germany; ^16^ Einheit für klinische Pharmakologie (EKLiP) Helmholtz Zentrum München ‐ German Research Center for Environmental Health Neuherberg Munich Germany

**Keywords:** immunotherapy, anti‐Hsp70 CAR NK cells, head and neck squamous cell carcinoma, membrane‐bound Hsp70, tumor‐associated antigen

## Abstract

An effective chimeric antigen receptor (CAR)‐based immunotherapy depends on both a suitable immune cell platform and a tumor‐specific antigen to overcome barriers in solid tumors. Natural killer (NK) cell lines are promising platforms for CAR constructs due to their inherent tumor‐killing ability, safety profile, and feasibility for standardized, off‐the‐shelf therapeutic use. Herein, four human NK cell lines (YT, KHYG1, NKL, and NK92) were retrovirally transduced with an anti‐Hsp70 CAR targeting membrane‐bound heat shock protein 70 (mHsp70), a tumor‐specific antigen with broad expression on many solid tumors, but not normal cells. Computational modeling suggested a strong binding between the CAR and the extracellular domain of mHsp70. Although all NK cell lines exhibited successful CAR integration and surface expression, only NKL and NK92 cells maintained stable CAR expression and long‐term viability. The anti‐Hsp70 CAR NKL and NK92 cells demonstrated enhanced expression of activation markers and secretion of cytotoxic effector molecules, and robust target‐specific killing of mHsp70‐positive cancer cells, while sparing mHsp70‐negative targets. Our findings validate the therapeutic potential of anti‐Hsp70 CAR NK cells and the suitability of NKL and NK92 cells for advancing off‐the‐shelf CAR NK cell therapies, thereby offering a promising strategy for targeting a broad range of solid tumors expressing mHsp70.

## Graphical abstract

This work presents a novel CAR NK cell platform targeting membrane‐bound Hsp70, a tumor‐specific antigen broadly expressed on solid tumors but not on normal cells. Computational modeling confirmed strong binding between the CAR construct and the Hsp70‐derived TKD peptide. The anti‐Hsp70 CAR NK cell lines (NKL, NK92) exhibited selective cytotoxicity against mHsp70‐positive cancer cells, with increased expression of activation markers and release of cytotoxic effector molecules. This figure was generated using illustrae.co as well as BioRender.com.

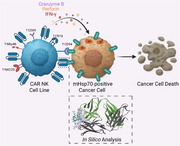

## 1 Introduction

Cellular immunotherapy has emerged as a sophisticated strategy for enhancing the intrinsic ability of the immune system to eradicate cancer cells [1]. Although initial therapeutic approaches utilizing non‐genetically modified immune cells demonstrated therapeutic potential, their clinical efficacy has been constrained by the tumor‐mediated attenuation of immune responses [2, 3]. Genetic modification of immune cells by incorporating synthetic receptors against tumor antigens offers a strategy to enhance antitumor activity and enable precise targeting of cancer cells [4, 5, 6].

Chimeric antigen receptors (CARs) are synthetic proteins that merge antigen‐recognition domains with those that trigger immune cell responses [7]. CAR T cells have delivered excellent efficacy in eliminating hematological malignancies, culminating in the FDA approval of CD19 CAR T cells [8]. However, broader application of CAR T cell therapies is hindered by their reliance on patient‐derived autologous cells to avoid the risk of graft‐versus‐host disease (GvHD) associated with allogeneic CAR T cells, even when T cells are derived from HLA‐matched donors [9, 10]. Generating autologous CAR T cells is costly and time‐consuming and may cause severe clinical complications such as cytokine release syndrome (CRS) [11, 12]. Consequently, there is a huge clinical need to identify safer, more efficient, and scalable cell products for CAR cell‐based therapeutics [13].

Natural killer (NK) cells can recognize and attack tumor cells without prior MHC‐restricted antigen stimulation by relying on a dynamic interplay between signaling via their activating and inhibitory surface receptors [14]. Like T cells, NK cells exert their antitumor effects through diverse mechanisms, including perforin‐ and granzyme‐mediated apoptosis, Fas ligand (FasL)‐mediated death receptor signaling, and tumor necrosis factor (TNF)‐related apoptosis‐inducing ligand (TRAIL). This is complemented by their ability to mediate antibody‐dependent cellular cytotoxicity (ADCC) via the low‐affinity Fc gamma receptor CD16 [15, 16]. Despite the versatility of their mechanisms of action, the activity of primary NK cells is limited in cancer patients by the immunosuppressive tumor microenvironment (TME), NK cell exhaustion, and reduced tumor‐killing capability [17, 18]. The CAR NK cell development has the potential to overcome these limitations. CAR NK cells have several advantages over CAR T cells, including a shorter in vivo lifespan, which reduces the risk of prolonged CAR expression‐associated complications such as autoimmunity and malignant transformation [19, 20, 21]. Allogeneic NK cells also carry a lower risk of GvHD, making them a safer therapeutic option [22]. Unlike CAR T cells, NK cells can target tumor cells HLA‐independently, potentially minimizing off‐tumor effects [23, 24]. Furthermore, NK cells predominantly secrete interferon‐gamma (IFN‐γ) and granulocyte‐macrophage colony‐stimulating factor (GM‐CSF), which are less likely to provoke a CRS [25, 26]. Preliminary clinical studies of CAR NK cell therapies in non‐solid tumors such as leukemia have shown encouraging results [13, 19, 27]. However, further research is required to optimize their application, particularly against solid tumors, where challenges such as limited cell persistence, poor tumor penetration, an immunosuppressive TME, and an impaired antigen presentation persist [13, 28, 29].

The selection of antigens to elicit tumor‐specific responses is a fundamental principle of advanced cancer immunotherapy. Heat shock protein 70 (Hsp70) is a cytosolic protein that resides in all nucleated cells and is highly overexpressed in the cytosol and, importantly, on the cell membranes of most solid tumors and hematological malignancies [30, 31, 32]. In cancer cells, a tumor‐specific lipid composition, including globotriaosylceramide (Gb3), enables anchorage of Hsp70 at the plasma membrane, a phenomenon not observed in normal cells [33]. This membrane Hsp70 (mHsp70) is a tumor‐specific antigen (TSA), and its expression is associated with advanced tumor stages, metastasis, therapeutic resistance, and a poor prognosis [34, 35, 36]. In addition, tumor cells actively release extracellular vesicles containing Hsp70, whose circulating levels reflect the mHsp70 expression status of cancer cells and correlate with tumor mass, grading, and resistance to therapy [37, 38].

We previously showed that incubating “exhausted” primary NK cells from tumor patients with a 14‐mer Hsp70 peptide TKDNNLLGRFELSG (“TKD”) and low‐dose interleukin (IL)‐2 stimulates their cytotoxicity against tumor cells expressing mHsp70 [39]. Preclinical studies, early‐phase clinical trials, and phase I/II clinical trials in patients with metastatic colorectal cancer and non–small cell lung cancer (NSCLC) have demonstrated that TKD/IL‐2‐primed NK cells can recognize and effectively kill mHsp70‐positive cancer cells in vivo and mediate beneficial clinical responses [31, 40, 41, 42], thereby validating mHsp70 as a clinically relevant target for cancer therapeutics [32]. To enhance the therapeutic potential of NK cells, we are developing and evaluating anti‐Hsp70 CAR approaches. As a previous proof‐of‐concept experiment, primary peripheral blood mononuclear cell (PBMC)‐derived T cells, which are unable to recognize mHsp70‐positive tumor cells, were genetically engineered with a CAR containing the mHsp70‐binding single‐chain variable fragment (scFv) domain derived from the cmHsp70.1 monoclonal antibody (mAb). Anti‐Hsp70 CAR T cells recognized and killed mHsp70‐positive colorectal cancer and head and neck squamous cell carcinoma (HNSCC) [4, 43]. These findings confirmed the potential of mHsp70 as an accessible TSA for developing novel targeted immunotherapies to improve overall treatment outcomes.

Inspired by our previous successful generation of anti‐Hsp70 CAR T cells [4], we engineered human NK cell lines with an mHsp70‐specific CAR, with the long‐term vision of developing an “off‐the‐shelf” CAR NK cell product. Our goal was to enhance the cytotoxicity of NK cell lines (YT, KHYG1, NKL, and NK92) against HNSCC tumor cells expressing high or low levels of mHsp70 and to characterize their target specificity and functional persistence. All cell lines were successfully transduced with anti‐Hsp70 CAR, but only NKL and NK92 maintained long‐term viability and stable CAR expression, and favorable responsiveness to TKD/IL‐2 priming. Anti‐Hsp70 CAR NKL and NK92 cells demonstrated increased secretion of IFN‐γ, granzyme B (GrB), and perforin, and improved cytotoxicity against human Cal27, UD‐SCC‐5, and SAS HNSCC cell lines expressing mHsp70. Notably, this response was antigen‐specific, as negligible cytotoxicity was observed against mHsp70‐negative human THP‐1 cells. Our results validate both the therapeutic relevance of Hsp70‐targeted CAR approaches and highlight the suitability of NKL and NK92 cells as effective platforms for anti‐Hsp70 CAR.

## 2 Results

### 2.1 mHsp70 Expression on HNSCC Cell Lines

**FIGURE 1:**
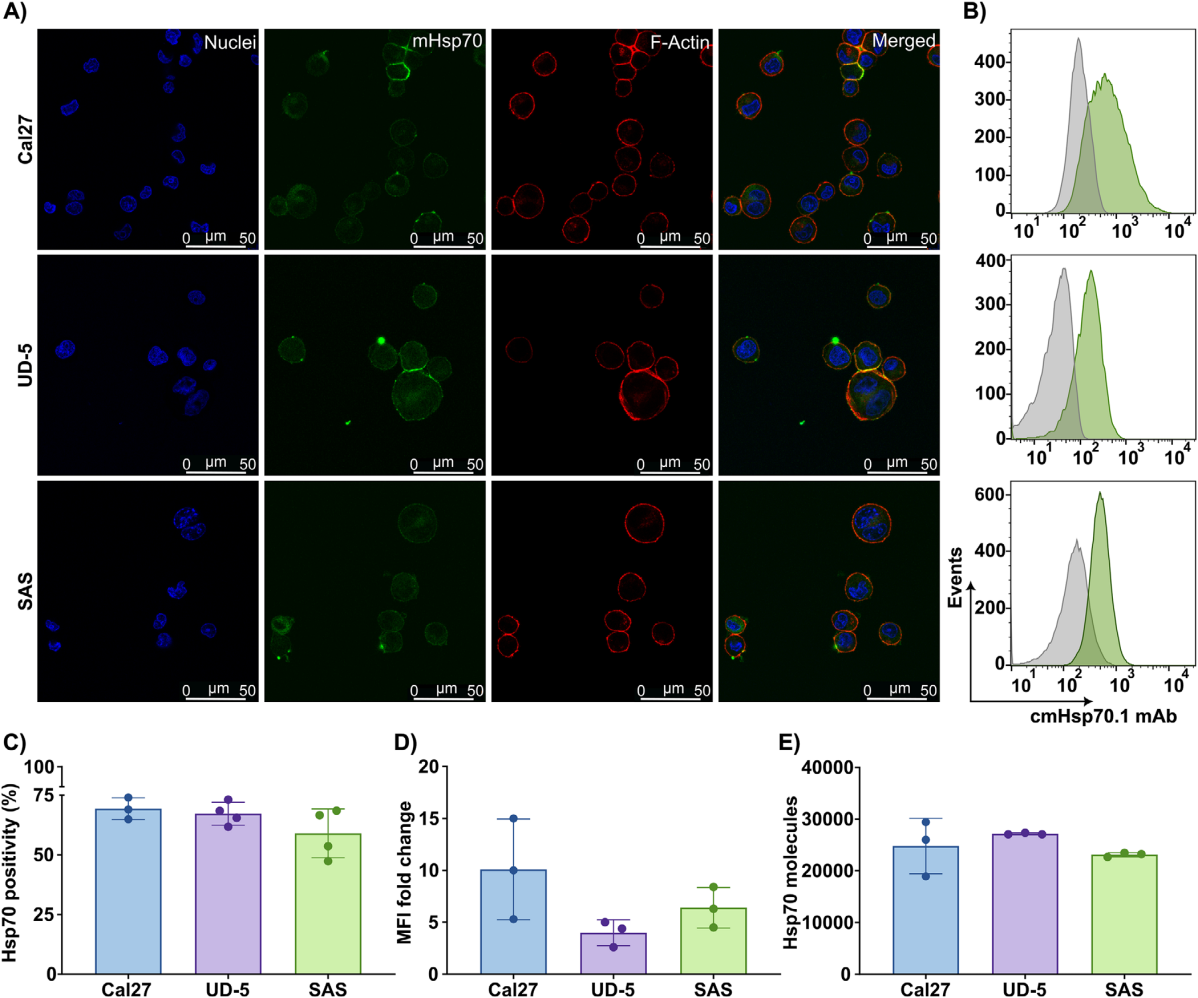
Membrane Hsp70 (mHsp70) expression on Cal27, UD‐5, and SAS HNSCC cell lines. (A) Representative confocal microscopy images of Cal27, UD‐5, and SAS cells stained with FITC‐cmHsp70.1 monoclonal antibody (mAb) to detect mHsp70 expression (green). The filamentous actin (F‐actin) cytoskeleton was visualized using Alexa Fluor Plus 647 Phalloidin (red), and nuclei were counterstained with Hoechst 33342 (blue). The co‐localization of red and green signals in the merged photomicrograph (yellow) demonstrates the localization of Hsp70 on the cell surface. The Scale bar is 50 µm. (B) Representative flow cytometric histograms using FITC‐cmHsp70.1 mAb to determine mHsp70 expression on HNSCC cell lines. Green histograms indicate mHsp70 expression, whereas gray histograms correspond to isotype‐matched controls (using mouse FITC‐IgG1 mAb). The mHsp70 expression is determined as (C) the percentage of mHsp70‐positive viable cancer cells and (D) MFI fold changes relative to the isotype control (mean ± SD, *n* ≥ three independent experiments; each dot represents an independent experiment). (E) Quantification of the number of Hsp70 molecules per viable cell in each cell line (mean ± SD, *n* ≥ 3 independent experiments; each dot represents an independent experiment).

CAR recognition efficiency largely depends on antigen type and its expression density on cancer cells [19]. Therefore, mHsp70 expression was determined on viable Cal27, UD‐5, and SAS HNSCC cell lines by confocal fluorescence microscopy (Figure 1A) and flow cytometry (Figure 1B–E).

Flow cytometric analysis revealed mHsp70 expression by approximately 69%, 67%, and 59% of Cal27, UD‐5, and SAS cells, respectively, with MFI values (fold change) of 10.1, 3.9, and 6.4 relative to the isotype control (Figure 1C,D). The gating strategy for detecting mHsp70 on cancer cells is shown in Figure S1. The absolute number of mHsp70 molecules per cell was determined using calibration beads: Cal27, 24,777 ± 5353; UD‐5, 27,153 ± 171; SAS, 23,113 ± 406 molecules per cell (Figure 1E). In summary, mHsp70 expression was detectable at relevant levels in all three cancer cell lines, validating their use as targets for anti‐Hsp70 CAR NK cells.

### 2.2 Molecular Docking and Molecular Dynamics (MD) Simulation Analysis of Hsp70‐Derived TKD Peptide Interactions With Chimeric scFv

Computational modeling and docking were used to predict the interaction between the Hsp70‐derived TKD peptide and the scFv domain of the anti‐Hsp70 CAR. The scFv model was generated from the cmHsp70.1 mAb sequence, with light and heavy chains modeled using high‐identity templates (100% and 88.7%, respectively; Figure S2A). Sequence alignments confirmed strong conservation of complementarity‐determining regions (CDRs), especially CDR‐H3 and CDR‐L1 (Figure S2B). These chains were assembled and docked using the HDOCK server, followed by energy minimization to generate a functional chimeric scFv structure (Figure S3A). Three independent 150 ns MD simulations of the *apo* scFv were performed to relax the model. Cluster analysis revealed three major conformational states based on backbone root mean square deviation (RMSD), with Cluster 2 being the most populated (Figure S3B–D). Representative structures from each *apo* cluster were used for TKD docking, and cluster 1 produced the most favorable binding energy (−206.74 kcal/mol) (Figure S4A). *Holo*‐state MD simulations similarly revealed three conformations, with Cluster 1 again showing the highest population and stability (Figure S4B–D). Structural snapshots highlighted conformational variability, particularly in CDR‐H3 (Figure S4E). This conformation, characterized by variability within CDR‐H3 (Figure S4E), was selected for all post‐MD analyses.

**FIGURE 2:**
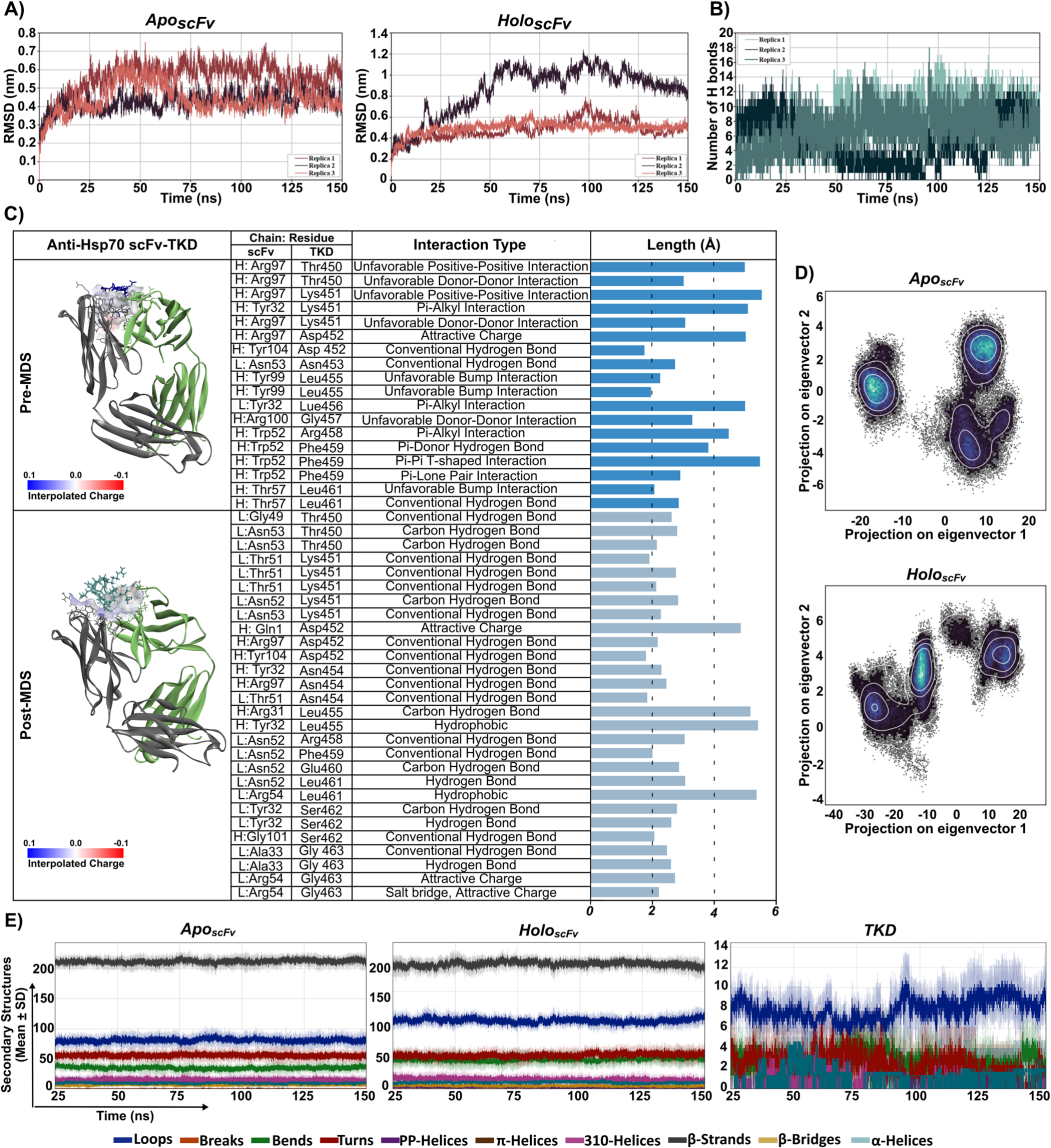
Molecular dynamics (MD) simulations of the interaction between Hsp70‐derived peptide (TKD) and anti‐Hsp70 scFv complex. MD simulations were performed to investigate the interaction between the TKD peptide and the scFv domain. The apo (scFv alone) and holo (TKD–scFv complex) systems underwent three independent 150 ns simulations. (A) Root mean square deviation (RMSD, in nm) of backbone atoms over time to indicate structural stability. (B) Time series of hydrogen bond (H‐bond) counts between TKD and scFv in the *holo* simulations. (C) Schematic 3D representations of TKD binding interactions with the scFv before and after simulation to show bond types and lengths (Å). Color scheme: green (scFv light chain), gray (scFv heavy chain), dark blue (pre‐MD TKD), and light blue (post‐MD TKD). An interpolated charge color bar is applied to the displayed surface only. Chains L and H refer to the light and heavy chains of the antibody, respectively. (D) Principal component analysis (PCA) projection showing backbone atomic motions along the first two principal components (PC1 and PC2) for the *apo* structure. (E) Time‐resolved secondary structure assignments via Dictionary of Secondary Structure of Proteins (DSSP) for *apo*, *holo*, and TKD structures, illustrating structural transitions throughout the simulations.

Following MD simulations, post‐MD analyses were conducted to evaluate stability, flexibility, and dynamics of the system. RMSD plots showed that the TKD‐scFv complex (*holo*) reached equilibrium more rapidly and exhibited lower overall fluctuations than the unbound scFv (*apo*) (Figure 2A). In contrast, the free TKD peptide remained moderately flexible during simulation, likely reflecting conformational adjustments required for stable binding (Figure S5A). Root Mean Square Fluctuation (RMSF) profiles further supported these observations, revealing reduced residue‐level fluctuations in the CDR loops upon TKD binding, particularly in the heavy chain (Figure S5B). Hydrogen bond analysis showed dynamic fluctuations ranging between 6 and 16 hydrogen bonds throughout the simulation, indicating persistent stability of the TKD–scFv complex (Figure 2B). Structural analysis indicated that TKD engages the CDR loops of the scFv through key interactions, including a hydrogen bond (Asn53–Lys451) and π–π stacking (Phe459–Trp52). Post‐MD simulations showed stable behavior in most runs, with reduced unfavorable contacts and the formation of new stabilizing hydrogen bonds (Figure 2C). Principal component analysis (PCA) revealed that TKD binding restricted the conformational space of the scFv, resulting in reduced large‐scale motions and increased structural rigidity. Comparative analysis indicated that peptide engagement induced the scFv to adopt distinct conformations not sampled in the apo state, reflecting a significant reorganization of its dynamic behavior (Figure 2D). Secondary structure analysis showed that TKD binding increased loop content in the scFv, primarily by reducing turn regions. The TKD peptide exhibited a heterogeneous secondary structure profile, comprising α‐helix, 3–10 helix, loops, and turns. Visual inspection highlighted conformational variability in loop regions, particularly within CDR‐H3 (Figure 2E). The findings demonstrate that Hsp70‐derived TKD peptide binding induces a stable, specific interaction with the anti‐Hsp70 scFv, as characterized by reduced flexibility, persistent hydrogen bonding, structural rigidity, and distinct conformational adaptations. To confirm the interaction suggested by our computational modeling, we assessed the binding affinity between the cmHsp70.1 mAb and full‐length recombinant Hsp70 protein using Microscale Thermophoresis (MST) (Figure S6). The measured dissociation constant (K_D_) of 1.91 nM reflects the strong affinity of cmHsp70.1 mAb for Hsp70 protein.

### 2.3 Transduction, Antigen Reactivity, and Phenotypic Characterization of Anti‐Hsp70 CAR NK Cells After TKD/IL‐2 Stimulation

**FIGURE 3:**
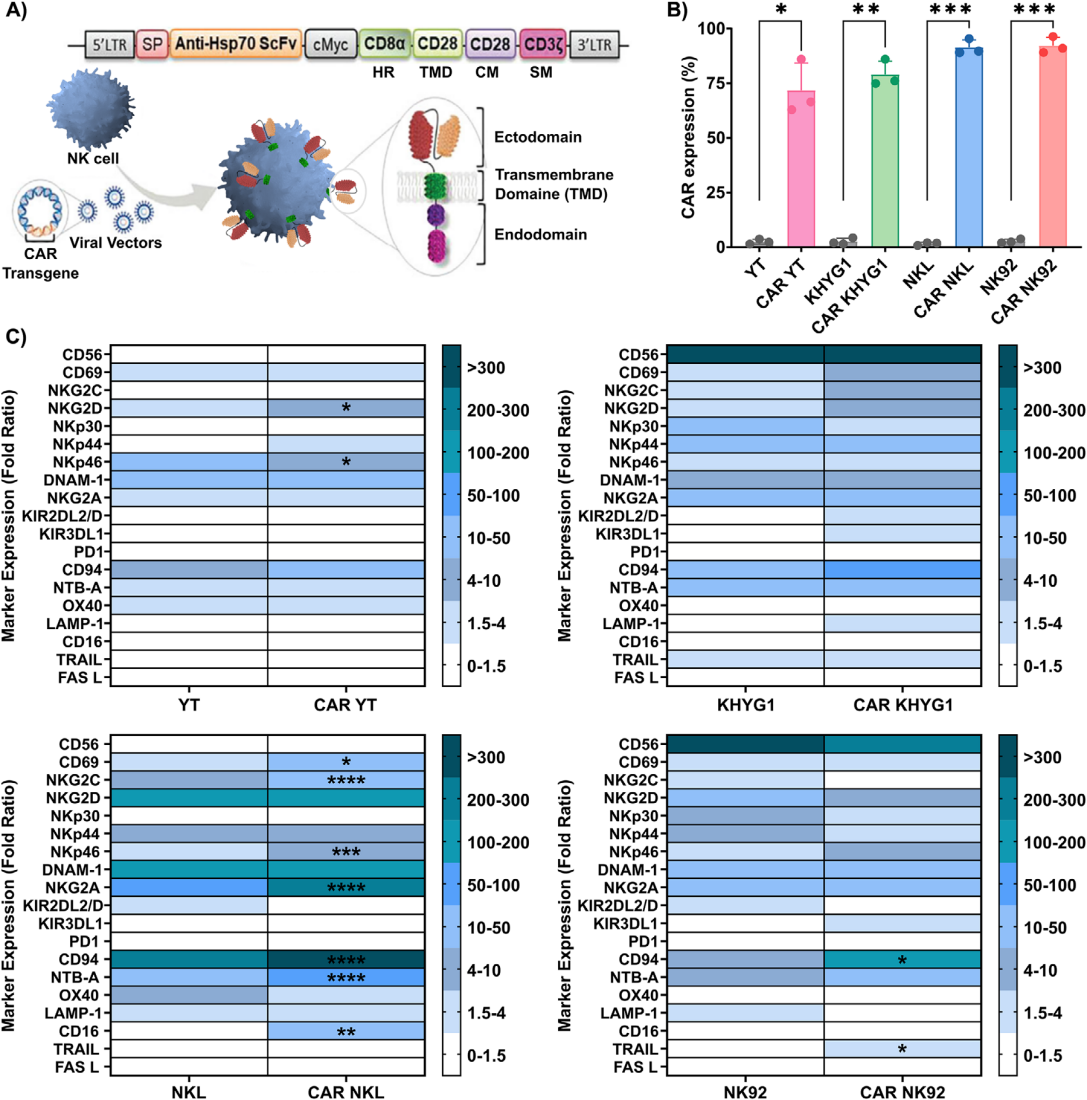
Generation and validation of anti‐Hsp70 CAR NK cell lines (YT, KHYG1, NKL, NK92) and phenotypic profiling following TKD/IL‐2 stimulation. (A) Schematic representation of the anti‐Hsp70 CAR construct and its retroviral transduction into NK cells. The construct consists of an ectodomain (including an immunoglobulin heavy‐chain signal peptide (SP), a single‐chain variable fragment (scFv) derived from the cmHsp70.1 mAb, and a c‐Myc tag, a CD8α hinge region (HR), a CD28 transmembrane domain (TMD), and an endodomain (including a CD28 co‐stimulatory moiety (CM) and a CD3ζ signaling moiety (SM)). (B) The transduction efficiency of the anti‐Hsp70 CAR was evaluated in transduced NK cell lines using flow cytometry. The assessment of the CAR expression and its binding capacity was performed upon incubation of either Unt or transduced NK cells with FITC‐labeled anti‐c‐Myc mAb and Alexa Fluor 647‐conjugated Hsp70 protein (50 µg/mL). An FITC‐labeled isotype‐matched control antibody and Alexa Fluor 647‐conjugated bovine serum albumin (BSA) were used to set the gating threshold for background fluorescence and nonspecific binding. The bar charts display the percentage of each CAR‐expressing NK cell line that is double‐positive for c‐Myc and bound Hsp70 protein in comparison to their Unt controls (mean ± SD, *n* ≥ 3 independent experiments; each dot represents an independent experiment). (C) The responsiveness of the CAR NK cells to Hsp70 stimulation was evaluated by quantifying the expression levels of surface activating and inhibitory NK cell receptors following incubation with Hsp70 peptide (TKD, 2 µg/mL) and low‐dose IL‐2 (160 IU/mL) at 5–7 days post‐transduction. Each CAR‐transduced and Unt NK cell line was analyzed after a 3‐day incubation with TKD/IL‐2 at 37°C. Receptor expression was evaluated using multiparameter flow cytometry. Results are calculated as fold‐change in MFI relative to isotype‐matched control groups and presented as the fold‐change ratio of stimulated cells to unstimulated cells (mean ± SD, *n* ≥ 3 independent experiments). Statistical significance is indicated as *p ≤ 0.05, **p ≤ 0.01, ***p ≤ 0.001, ****p ≤ 0.0001.

To enhance the potential of NK cell lines to recognize and kill tumor cells expressing mHsp70, YT, KHYG1, NKL, and NK92 cells were transduced with a previously reported anti‐Hsp70 CAR construct [4]. The CAR cassette was engineered by integrating an scFv derived from the cmHsp70.1 mAb connected via a CD8α hinge region (HR) to the transmembrane domain (TMD) of CD28, with an intracellular CD28 co‐stimulatory moiety (CM) and a CD3ζ signaling moiety (SM) (Figure 3A). Retroviral vectors were used to transduce NK cells with anti‐Hsp70 CAR, and transduction efficiency was determined by flow cytometry. The analysis revealed that a large proportion of each CAR‐transduced NK cell line was positive for both CAR expression and Hsp70 binding ability. Transduction efficiencies for anti‐Hsp70 CAR NK cells exceeded 70% across all tested NK cell lines (Figure 3B).

Next, expression of activating and inhibitory receptors, co‐receptors, and markers associated with cytotoxic activity was flow cytometrically compared on both anti‐Hsp70 transduced CAR NK cell lines and their untransduced (Unt) counterparts in response to stimulation with Hsp70 peptide and low‐dose IL‐2. Cells were profiled for the expression of the classical NK cell marker NCAM (CD56), activating receptors (CD69, NKG2C, NKG2D, NKp30, NKp44, NKp46, and DNAM‐1), inhibitory receptors (NKG2A, KIRs, and PD‐1), co‐stimulatory receptors (CD94, NTB‐A, and OX40), the degranulation marker LAMP‐1/CD107a, the ADCC mediator CD16, and cytotoxic ligands (TRAIL and FasL) (Figure 3C). Representative dot plots of the gating strategy for the NK cell immunophenotyping are provided in Figure S7.

Priming with TKD/IL‐2 shifts the balance between activating and inhibitory receptors, with a tendency to increase densities of activating receptors in both Unt and CAR‐transduced NK cells. Notably, NK cell activation by TKD/IL‐2 correlates with increased CD94 expression, independent of transduction status. This result aligns with previous findings of our group and highlights the critical role of the heterodimeric C‐type lectin receptor CD94 in the interaction with mHsp70 on tumor cells [39, 44]. Moreover, anti‐Hsp70 CAR NKL cells (****p ≤ 0.0001) and anti‐Hsp70 CAR NK92 cells (*p ≤ 0.05) exhibited a higher CD94 expression density compared with Unt cells (Figure 3C). The most pronounced phenotypic changes occurred in the NKL cell line after CAR transduction, with higher expression of CD69, NKG2C, NKp46, NKG2A, NTBA, CD16, and CD94.

### 2.4 Stability of Anti‐Hsp70 CAR Expression in Viable CAR NK Cell Lines

**FIGURE 4:**
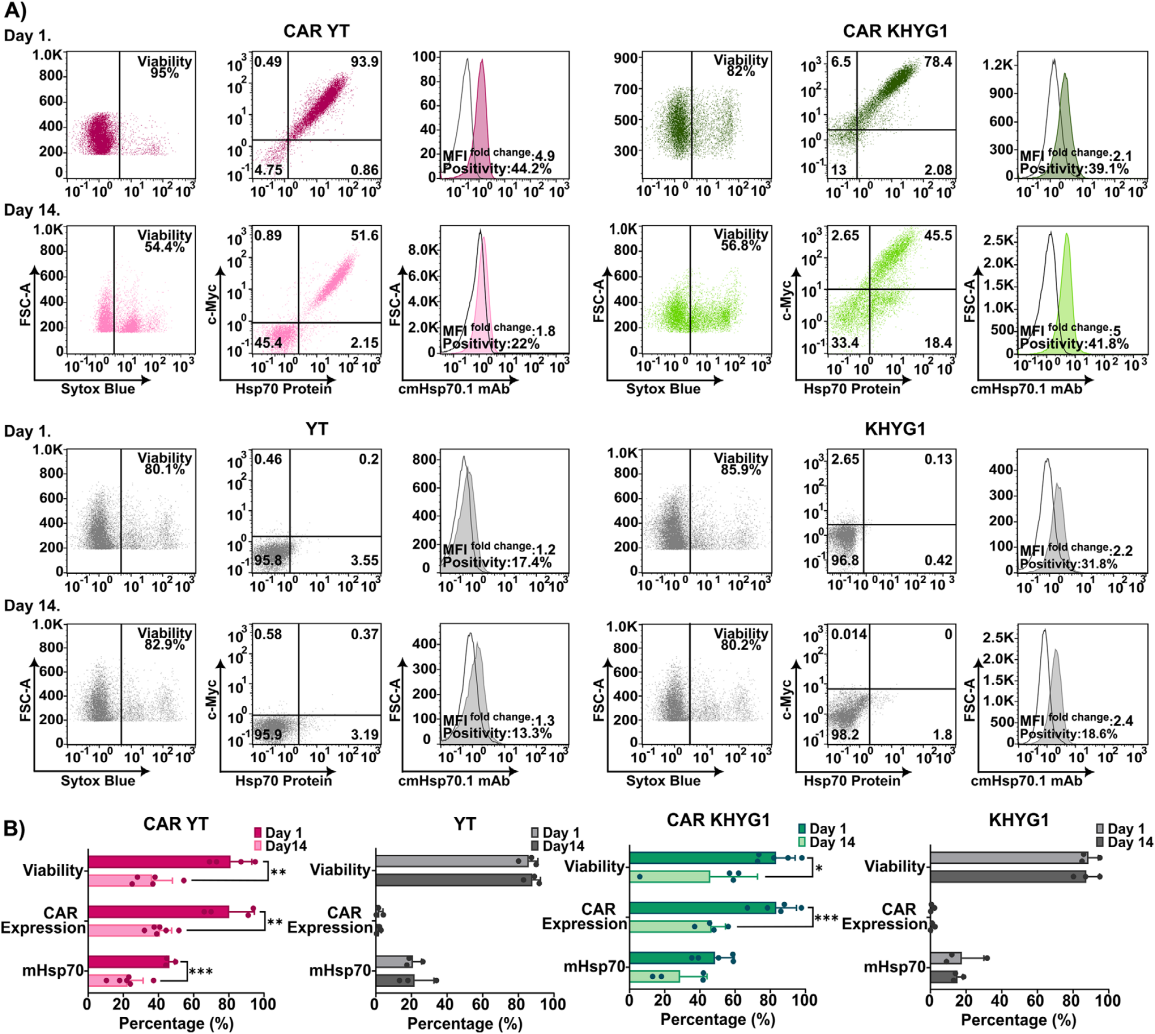
Expression and persistence of anti‐Hsp70 CAR in transduced YT and KHYG1 NK cell lines. Flow cytometric analysis was performed to evaluate the expression of anti‐Hsp70 CAR in viable transduced KHYG1 and YT cells by detecting dual positivity for FITC‐c‐Myc tag expression and bound Alexa Fluor 647‐labeled Hsp70 protein. Cell viability was assessed by gating live cells using SYTOX Blue viability dye. The expression of mHsp70 was evaluated using cmHsp70.1 mAb. The assessments were performed on days 1 and 14 post‐sorting. Unt cells were tested in parallel as controls for comparison. (A) Representative scatter plots from flow cytometric analysis of anti‐Hsp70 CAR‐transduced and Unt YT and KHYG1 cells. (B) The level of viability, anti‐Hsp70 CAR expression, and mHsp70 expression of transduced and Unt cells on days 1 and 14 post‐sorting. All data are presented as percentages (%) relative to isotype‐matched control (mean ± SD, *n* ≥ 3 independent experiments; each dot represents an individual measurement). Statistical significance is indicated as *p ≤ 0.05, **p ≤ 0.01, ***p ≤ 0.001.

All anti‐Hsp70 CAR NK cell lines were enriched by cell sorting for CAR positivity before a series of quality control assessments. Cell viability, CAR expression stability, and binding to fluorescence‐labeled Hsp70 protein were rechecked in the sorted, transduced NK cell lines by flow cytometry on days 1 and 14 post‐sorting. A representative flow cytometric analysis indicating cell viability, Hsp70 binding capacity, and mHsp70 expression on CAR‐transduced and Unt YT and KHYG1 cell lines is shown in Figure 4A.

As depicted in Figure 4B, anti‐Hsp70 CAR‐transduced YT and KHYG1 cells showed good viability and high CAR expression (>80%) on day 1 post‐sorting. However, on day 14, both viability and CAR expression declined to 36.4 ± 11.5% (YT viability, **p ≤ 0.01), 40.8 ± 6.6% (YT CAR, **p ≤ 0.01), 45.9 ± 26.8% (KHYG1 viability, *p ≤ 0.05), and 46.6 ± 7.8% (KHYG1 CAR, ***p ≤ 0.001). In contrast, no changes were observed in the viability of Unt YT and KHYG1 cells between days 1 and 14. Interestingly, CAR‐engineered YT and KHYG1 cells showed an increased percentage of mHsp70‐positive cells (46.2 ± 3% and 48.6 ± 10.9%, respectively) on day 1 compared with Unt controls, which then dropped over time. This finding indicates that viral transduction induces mHsp70 on YT and KHYG1 cells. Overall, CAR‐transduced YT and KHYG1 cell lines demonstrated reductions in CAR expression paralleled by a drop in cell viability between day 1 and day 14, along with a decline in the proportion of mHsp70‐positive cells.

**FIGURE 5:**
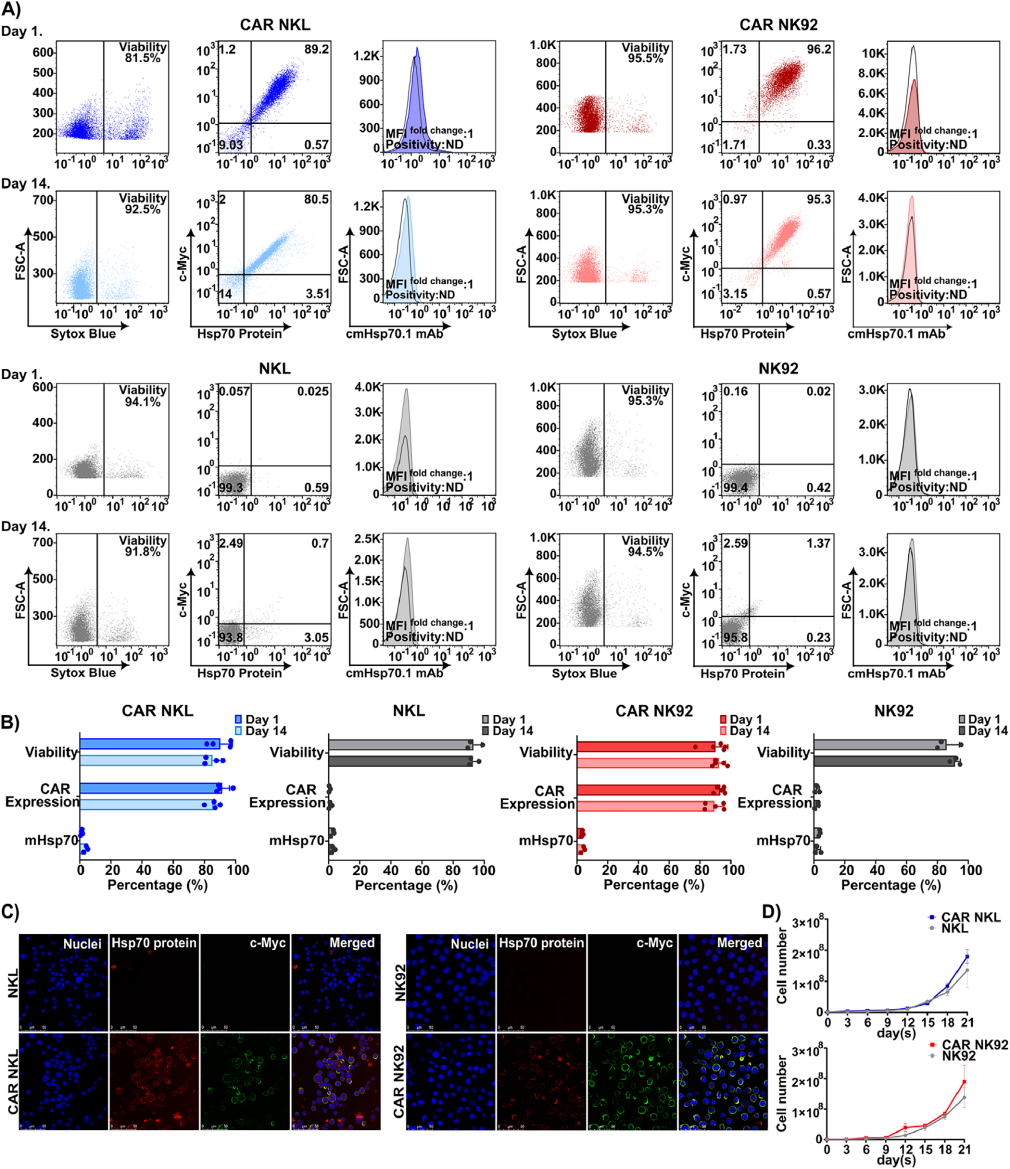
Expression and persistence of anti‐Hsp70 CAR in transduced NKL and NK92 cell lines. Analysis and assays were performed as described in the legend of Figure 4. (A) Representative scatter plots from flow cytometric analysis of anti‐Hsp70 CAR‐transduced and Unt NKL and NK92 cells on days 1 and 14 post‐sorting. (B) The level of cell viability, anti‐Hsp70 CAR expression, and mHsp70 expression of transduced and Unt cells on days 1 and 14 post‐sorting. All data are presented as a percentage (%) relative to isotype‐matched control (mean ± SD, *n* ≥ 3 independent experiments; each dot represents an individual measurement). (C) Anti‐Hsp70 CAR expression on transduced NKL and NK92 cells visualized by confocal microscopy using Alexa Fluor 647‐labeled Hsp70 protein (red) and FITC‐conjugated anti‐c‐Myc mAb (green). Nuclei were stained with Hoechst 33342 (blue). The Unt cells were also imaged as a negative control. The scale bar is 50 µm. (D) The expansion potential of CAR‐transduced and Unt NK cells was evaluated in parallel cultures. Following sorting, the cells were seeded at an initial density of 5 × 10^5^ cells per well, with viable cell counts measured every three days over 21 days (mean ± SD, *n* ≥ 3 independent experiments).

This observation led us to hypothesize that fratricide, triggered by the recognition of mHsp70 on anti‐Hsp70 CAR‐transduced cells, followed by adaptation to chronic mHsp70 exposure, may drive a regulatory response that contributes to the gradual reduction in the CAR expression over time. To explore this hypothesis, we also compared CAR‐transduced and Unt NKL and NK92 cell lines (Figure 5). Both anti‐Hsp70 CAR‐transduced NKL and NK92 cell lines and their Unt counterparts showed a comparably high viability, a nearly undetectable mHsp70 expression, and a stable high anti‐Hsp70 CAR expression on day 1 and day 14 (Figure 5A,B). The expression of anti‐Hsp70 CAR was visualized by examining the co‐localization of fluorescently labeled anti‐c‐Myc mAb (green) and Hsp70 protein (red) on the surface of CAR‐transduced NKL and NK92 cells. The alignment of green and red signals led to yellow fluorescence, whereas no green or red signals were observed on the surface of Unt cells (Figure 5C). This colocalization indicates that the CAR is not only expressed but also capable of engaging Hsp70 on the cell surface, consistent with our previous findings demonstrating functional Hsp70 recognition when the same CAR construct was introduced into T cells [4]. Additionally, we compared the growth rates of anti‐Hsp70 CAR NKL and NK92 cells and their Unt counterparts over a 21‐day culture period. The experiment was initiated with 5 × 10^5^ viable cells per group post‐sorting, and cell numbers were assessed every three days. As illustrated in the cell expansion curves (Figure 5D), both anti‐Hsp70 CAR NK cell lines and their corresponding Unt cells displayed a similar proliferation behavior over 21 days. The doubling time was calculated as 2.11 ± 0.18 days for anti‐Hsp70 CAR NKL cells and 2.48 ± 0.23 days for anti‐Hsp70 CAR NK92 cells. In summary, NKL and NK92 cells provide ideal acceptor cells for anti‐Hsp70 CAR transduction due to stable and high CAR expression over time, favorable expansion rates, and the lack of mHsp70 expression after CAR transduction as a potential target for fratricide.

### 2.5 Cytokine Release by Anti‐Hsp70 CAR NK Cells Following Hsp70 Peptide Priming or Exposure to Cancer Cells Expressing mHsp70

**FIGURE 6:**
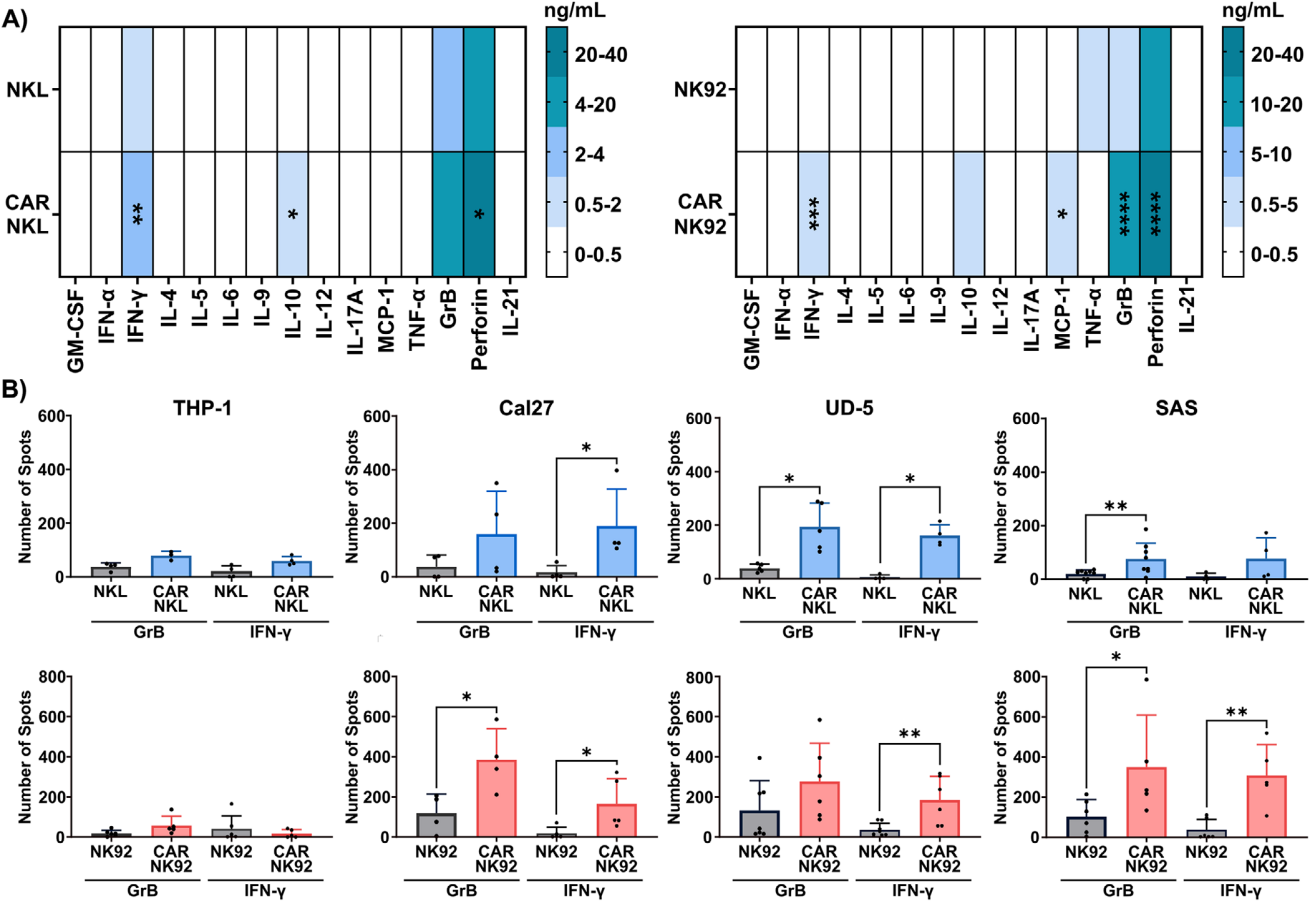
Immune response profiles of anti‐Hsp70 CAR NKL and NK92 cells. (A) Multiplex cytokine and chemokine levels in the supernatant of anti‐Hsp70 CAR NKL and NK92 cell cultures were quantified by flow cytometry at 72 h after stimulation with TKD/IL‐2. The Unt NK cells (NKL and NK92) were used as controls for comparison (mean ± SD, *n* ≥ 3 independent experiments). (B) Levels of GrB and IFN‐γ released by effector cells after a 6‐h co‐culture with target cells. Effector (anti‐Hsp70 CAR NKL or CAR NK92) and target (THP‐1, Cal27, UD‐5, and SAS) cells were co‐cultured at an effector‐to‐target (E:T) ratio of 1:1, and the release of GrB and IFN‐γ was measured using a double‐color ELISpot assay. Data are presented as spots per well (mean ± SD, *n* ≥ 4 independent experiments, each performed in duplicate; each dot represents the mean of one independent experiment). Statistical significance is indicated as *p ≤ 0.05, **p ≤ 0.01, ***p ≤ 0.001, ****p ≤ 0.0001.

Targeting mHsp70 on cancer cells by NK cells leads to the secretion of effector molecules involved in cell‐mediated cytotoxicity and inflammatory responses. Our previous studies have shown differential secretion patterns in unstimulated and TKD/IL‐2‐stimulated primary NK cells, particularly for TNF‐α, IFN‐γ, and lytic granules such as GrB and perforin [39]. Herein, we assessed whether the secretion profiles of anti‐Hsp70 CAR NKL and NK92 cells differ from those of their corresponding Unt counterparts following TKD/IL‐2 stimulation (Figure 6). The data revealed a higher secretion of IFN‐γ, GrB, and perforin in the supernatants of TKD/IL‐2‐stimulated anti‐Hsp70 CAR‐transduced NK cells compared with Unt cells (Figure 6A). Specifically, anti‐Hsp70 CAR NKL showed an increased secretion of IFN‐γ (**p ≤ 0.01) and perforin (*p ≤ 0.05), whereas anti‐Hsp70 CAR NK92 cells demonstrated a more pronounced increase in IFN‐γ (***p ≤ 0.001), GrB (****p ≤ 0.0001), and perforin (****p ≤ 0.0001) secretion (Figure 6A). Additionally, production of cytokines such as IL‐10 by anti‐Hsp70 CAR NKL cells (*p ≤ 0.05) and MCP‐1 in anti‐Hsp70 CAR NK92 cells (*p ≤ 0.05) was higher compared with Unt cells.

Findings were validated in a 6‐h co‐culture experiment of effector and target cells expressing various levels of mHsp70, followed by measurement of NK cell‐mediated GrB and IFN‐γ secretion (Figure 6B). The results showed that anti‐Hsp70 CAR NKL and NK92 cell lines induced a more robust GrB release than their Unt counterparts (Figure 6B). A similar pattern was also observed for IFN‐γ secretion, with a significantly higher FluoroSpot response for both anti‐Hsp70 CAR NK cell platforms than Unt cells. Interestingly, the secretion profile in each co‐culture condition varied by cancer and NK cell type. For example, enhanced IFN‐γ release was significant in co‐cultures of anti‐Hsp70 CAR NKL cells with Cal27 (*p ≤ 0.05) and UD‐5 (*p ≤ 0.05) cells, whereas GrB release was more prominent in co‐cultures with UD‐5 (*p ≤ 0.05) and SAS (**p ≤ 0.01) cells. For anti‐Hsp70 CAR NK92 cells, GrB release was increased in co‐cultures with Cal27 (*p ≤ 0.05) and SAS (*p ≤ 0.05) cells, whereas IFN‐γ release was notably increased in co‐cultures with Cal27 (*p ≤ 0.05), UD‐5 (**p ≤ 0.01), and SAS (**p ≤ 0.01) cells. Anti‐Hsp70 CAR NKL and NK92 cells displayed a consistently increased GrB and IFN‐γ release in response to co‐incubation with cancer cells expressing high levels of mHsp70, including Cal27 and UD‐5 cells (Figure 6B). In contrast, no detectable secretion of GrB or IFN‐γ was observed after a co‐culture with THP‐1 cells, which do not express mHsp70 (Figure S8), thereby confirming the target specificity of anti‐Hsp70 CAR NK cells (Figure 6B). Representative ELISpot images illustrating GrB and IFN‐γ secretion by both transduced and Unt NKL and NK92 cells after co‐culture with THP‐1 and Cal27 are provided in Figure S9. Taken together, ELISpot analysis of GrB and IFN‐γ release supports the functional targeting of mHsp70 by anti‐Hsp70 CAR NKL and NK92 cells and demonstrates that their responses correlate with mHsp70 expression by HNSCC cells.

### 2.6 Functional Activity of Anti‐Hsp70 CAR NK Cells Against mHsp70‐Positive Cancer Cells

**FIGURE 7:**
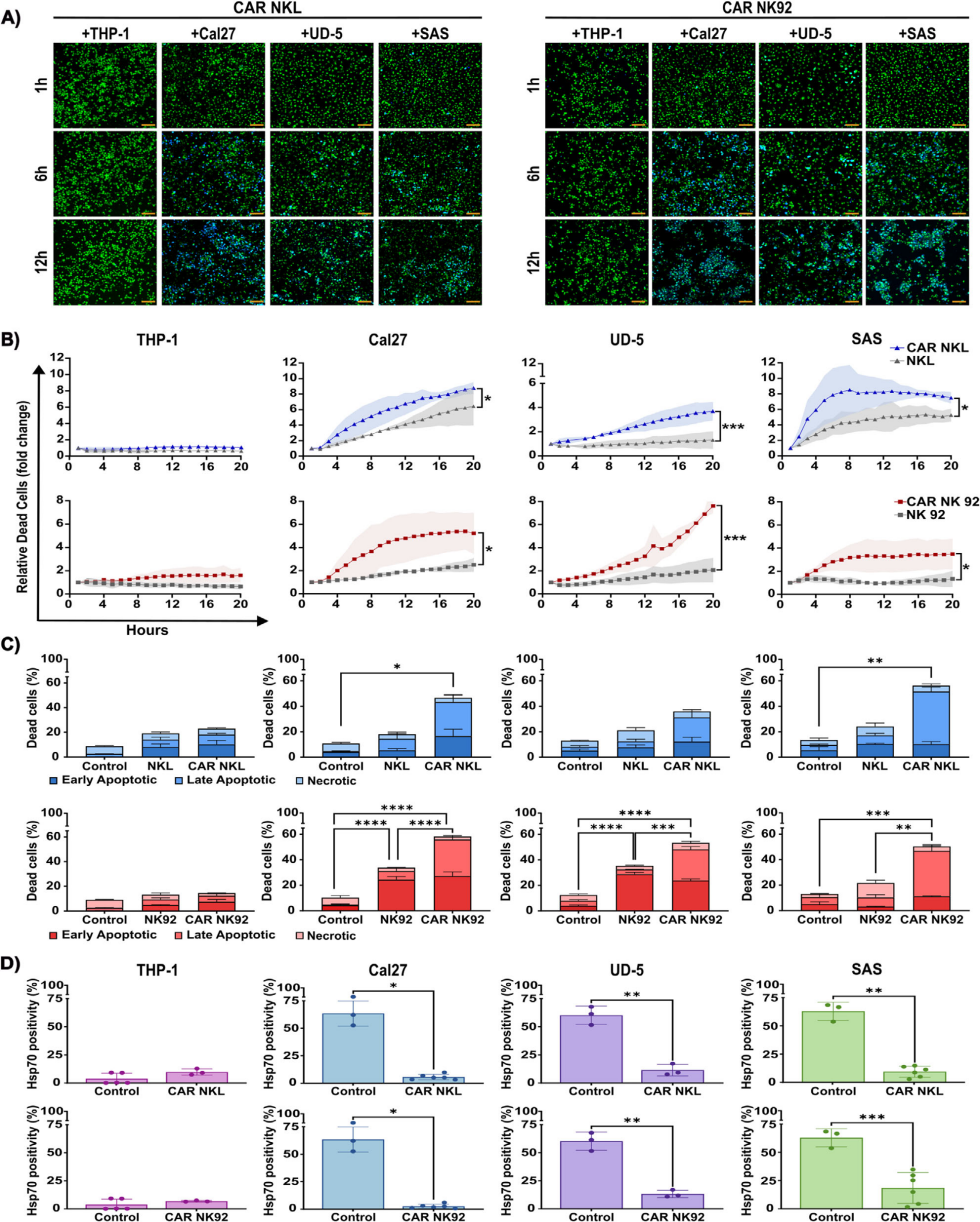
Anti‐Hsp70 CAR NK cell‐mediated cytotoxicity against mHsp70‐positive tumor cells. The anti‐Hsp70 CAR NKL and CAR NK92 cells were co‐incubated with Cal27, UD‐5, or SAS HNSCC target cells at an E:T ratio of 1:1 for 20 h. Unt NKL and NK92 effector cells, and THP‐1 target cells were used to assess the baseline cytotoxicity and specificity of the anti‐Hsp70 CAR NK cell activity, respectively. (A) Representative time‐lapse fluorescence microscopy images of target cells co‐cultured with anti‐Hsp70 CAR NKL cells (left) and anti‐Hsp70 CAR NK92 cells (right). Monitoring was performed for 20 h, and images captured at 1, 6, and 12 h after co‐incubation are shown. Target cells were pre‐labeled with PKH67 (green), and SYTOX Blue was used to indicate dead cells (blue). The labeled NK cells were excluded from the images to allow better visualization of the dynamic changes in the cancer cells. The scale bar is 100 µm. (B) Tumor cell death quantified over a 20‐h time course. Relative dead cell counts were normalized to the zero‐time point, and the comparisons were made between their co‐cultures with anti‐Hsp70 CAR NKL cells (blue lines) or anti‐Hsp70 CAR NK92 cells (red lines), and their Unt counterparts (gray) (mean ± SD, *n* ≥ 3 independent experiments). (C) Flow cytometric analysis of apoptosis and necrosis in cancer cells after a 6‐h co‐culture with effector cells using Annexin V‐FITC/PI staining. Untreated target cells served as control for each condition (mean ± SD, *n* ≥ 3 independent experiments). (D) Flow cytometric analysis of the positivity of mHsp70‐expressing viable tumor cells performed after a 6‐h co‐culture with either anti‐Hsp70 CAR NKL cells (top row) or anti‐Hsp70 CAR NK92 cells (bottom row). Untreated target cells served as control (mean ± SD, *n* ≥ 3 independent experiments; each dot represents an independent experiment; *n* = 3 are paired data). Statistical significance is indicated as *p ≤ 0.05, **p ≤ 0.01, ***p ≤ 0.001, ****p ≤ 0.0001.

Live‐cell imaging was employed to visualize cytolytic activity of anti‐Hsp70 CAR NKL and CAR NK92 cells against cancer cells. Images captured at 1, 6, and 12 h are provided as representative examples of the dynamic changes in cancer cell viability (Figure 7A). In parallel, cancer cells were co‐cultured with the corresponding Unt NK cells over the same period, and the representative images are provided in Figure S10. Cytolytic activity was quantified over time by measuring the accumulation of SYTOX Blue‐positive cells within the cancer cell populations (Figure 7B). Quantification of cell death revealed no killing of THP‐1 cells by either anti‐Hsp70 CAR NKL or NK92 cells compared with their respective Unt cells (Figure 7B). In contrast, a clear time‐dependent increase in the proportion of SYTOX Blue‐positive dead cells was observed in Cal27 (*p ≤ 0.05), UD‐5 (***p ≤ 0.001), and SAS (*p ≤ 0.05) cell lines after co‐incubation with both anti‐Hsp70 CAR NKL or CAR NK92 cells in comparison to Unt NK cell controls (Figure 7B).

Annexin V/PI staining after a 6 h co‐incubation demonstrated efficient cell killing of the mHsp70‐positive Cal27, UD‐5, and SAS cells by both anti‐Hsp70 CAR NKL cells and CAR NK92 cells (Figure 7C). Although Unt NK cells induced some apoptosis, anti‐Hsp70 CAR NK cells triggered significantly higher levels of cell death. Notably, mHsp70‐positive HNSCC cells (Cal27, UD‐5, SAS) were more susceptible to CAR NK cell‐mediated cytotoxicity than mHsp70‐low THP‐1 cells. However, the level of apoptosis of mHsp70‐expressing cancer cells appeared to depend on the individual cell line. Cal27 and UD‐5 cells displayed similar levels of early and late‐stage apoptosis, whereas the majority of SAS cells progressed to late apoptosis. The cytotoxic potential of anti‐Hsp70 CAR‐transduced and Unt KHYG1 and YT cell lines was also tested against different HNSCC cells and THP‐1 cells. As expected, no significant difference in killing ability was observed between the CAR‐transduced and Unt KHYG1 and YT NK cells across the tested cancer cell types (Figure S11). These findings further reflect the regulatory role of CAR transduction in enhancing NK cell‐mediated antitumor activity. The gating strategy is shown in Figure S12.

Next, we evaluated the mHsp70 expression on viable cancer cells following a 6 h co‐incubation with anti‐Hsp70 CAR NKL and CAR NK92 cells to confirm that cytotoxicity was driven by mHsp70 expression (Figure 7D). THP‐1 cells served as a negative control target and consistently remained mHsp70‐negative over time. A relevant reduction in mHsp70‐expressing Cal27 (*p ≤ 0.05), UD‐5 (**p ≤ 0.01) and SAS (**p ≤ 0.01 upon co‐incubation with anti‐Hsp70 CAR NKL; ***p ≤ 0.001 upon co‐incubation with CAR NK92) cells was observed, supporting the proposition that anti‐Hsp70 CAR NK cells predominantly eliminate tumor cells expressing mHsp70, resulting in a remaining cancer cell population with a low or absent mHsp70 expression. Overall, these findings support the specificity of anti‐Hsp70 CAR NK cell lines for mHsp70‐expressing tumor cells.

## 3 Discussion

Given the resistance mechanisms in solid tumors that limit current cell‐based therapies, efforts have focused on boosting the antitumor activity of immune cells. In this context, CAR technology improves cell‐based immunotherapies by genetically engineering immune cells such as T and NK cells for targeted tumor killing [45]. Building upon our earlier work demonstrating that mHsp70 CAR T cells can kill mHsp70‐positive tumor cells [4], we engineered NK cell lines using retroviral vectors bearing an anti‐Hsp70‐specific CAR to enhance recognition and elimination of mHsp70‐positive tumors. The effectiveness and safety of CAR NK cell therapies depend on selecting a TSA with no or minimal expression in healthy tissues, and favorable receptor–ligand interactions, which are critical for optimal CAR signaling and function [46]. The mHsp70, expressed on many tumors but not on normal cells, is a compelling candidate that reduces the risk of on‐target/off‐tumor effects [32]. Such effects have limited other CAR therapies, for example, those targeting HER‐2/neu due to expression on vital healthy tissues [47], or CD19, which results in depletion of normal B cells [48]. HNSCC tumors frequently exhibit elevated stress responses reflected in overexpression of cytosolic and membrane‐bound stress proteins, including mHsp70 [49, 50, 51]. The experimental workflow is summarized in Figure S13. Our findings underscore the therapeutic potential of anti‐Hsp70 CAR NK cells and position them as an alternative or complement to CAR T‐cell therapies for treating mHsp70‐positive malignancies [4]. Notably, CAR NK cells do not require HLA matching or labor‐intensive isolation of patient‐derived T cells, and typically induce milder cytokine release, supporting their “off‐the‐shelf” use in clinical settings [13].

Our computational analysis presents an atomic‐resolution model of an antibody recognizing the TKD peptide from the C‐terminal region of Hsp70. Although this Hsp70‐derived peptide is well established for stimulating primary NK cells via the CD94 complex and enhancing immune responses against mHsp70‐positive tumors [39, 52], its structural interactions with adaptive immune receptors, such as antibodies, have not been previously explored computationally. Our modeling demonstrated a stable and energetically favorable interaction between the TKD peptide and the antigen‐recognition site of a chimeric antibody. Collectively, these results support a high‐affinity interaction, positioning the peptide as a competent epitope for antibody recognition and as a promising therapeutic target.

Maintaining stable CAR expression is essential, as stability can be affected by multiple factors [19]. These include tonic signaling in the absence of ligand [53], sustained antigenic engagement [54], transcriptional repression through epigenetic modifications [55], self‐antigen‐driven fratricide and self‐recognition [56], ligand‐induced receptor endocytosis and lysosomal degradation [57], suboptimal CAR design [58], cellular stress responses [59], and immunogenicity of non‐human CAR components [60]. In this study, we investigated the stability of the anti‐Hsp70 CAR and its impact on viability across four NK cell lines. These analyses provide insights into how mHsp70 expression on host NK cells influences CAR persistence and immune cell fitness post‐transduction. The observed decline in CAR expression and viability in anti‐Hsp70 CAR‐transduced YT and KHYG1 cells during prolonged culture suggests self‐recognition. We hypothesize that fratricide may occur, wherein anti‐Hsp70 CAR NK cells recognize and eliminate neighboring cells expressing mHsp70 [61]. Additionally, chronic ligand exposure may lead to CAR downregulation, possibly through antigen modulation or selective pressure favoring cells with lower mHsp70 expression [54, 62]. A similar phenomenon has been seen in CAR T cells exposed to prolonged antigen stimulation, where CAR downregulation can result from transcriptional silencing, epigenetic reprogramming, or receptor degradation [63, 64]. Such changes are reminiscent of NK cell exhaustion or CAR internalization under persistent antigen pressure [65]. Fratricide has previously been documented for CD38 CAR NK cells, where ex vivo expansion enhances CD38 expression and triggers self‐targeting [66]. Similarly, CD33 expression can be induced under certain conditions, such as cytokine stimulation or during ex vivo expansion, increasing the risk of fratricide in CD33 CAR NK therapies [67]. Because our NK cell lines originate from malignant cells, their potential expression of mHsp70 is unfavorable, as it may promote self‐recognition and fratricide, contributing to the selective CAR loss in YT and KHYG1. To address these limitations, approaches, including CRISPR/Cas9‐mediated genetic engineering of NK cells [68, 69] and transient CAR expression [70], could be explored to optimize CAR NK cell function and mitigate the risk of self‐targeting. In our study, anti‐Hsp70 CAR NKL and NK92 cells, which do not express mHsp70 upon CAR‐transduction, maintained stable CAR expression and viability over time.

To investigate how the Hsp70‐targeting CAR construct enhances NK cell stimulation, we examined receptor expression profiles after priming with Hsp70 peptide TKD and IL‐2. This approach, previously shown to mimic tumor‐associated stress conditions, promotes NK cell activation by inducing stress‐related ligand expression [39]. Anti‐Hsp70 CAR NK cells exhibited greater upregulation of CD94 and additional activating receptors following TKD/IL‐2 stimulation compared with Unt counterparts [39, 52]. Moreover, NKL cells showed increased surface expression of NKG2A, an inhibitory receptor that may reflect a feedback mechanism to limit overactivation and cytotoxic damage, which is consistent with a safer treatment strategy [71]. Importantly, NKL cells showed the most pronounced phenotypic shift after anti‐Hsp70 CAR introduction, highlighting the importance of compatible cell platforms for an optimized CAR therapy. These results indicate that the engineered anti‐Hsp70 CAR retained antigen specificity and enhanced the immunoreactivity of transduced NK cells.

Anti‐Hsp70 CAR NK cells exhibit enhanced functional activity, particularly through the secretion of the serine protease GrB and perforin, both key cytolytic effectors in target cell lysis [4, 72]. Consistently, a greater level of IFN‐γ, a marker of apoptosis‐mediated antitumor activities, was observed in CAR‐transduced NK cells compared with parental cells [73]. There were no noteworthy differences between NK cell‐associated cytokine release patterns in the supernatant, with both anti‐Hsp70 CAR NKL and NK92 cell lines showing similar trends. Beyond cytotoxic granule secretion, we also detected immunomodulatory cytokines, with IL‐10 present in both anti‐Hsp70 CAR NK cell types and MCP‐1 predominantly expressed by anti‐Hsp70 CAR NK92 cells. The selective upregulation of this chemokine may represent a feedback mechanism modulating the inflammatory microenvironment or promoting immune cell recruitment [74]. These findings suggest that CAR engineering not only boosts direct cytotoxicity but also reshapes the broader immunoregulatory profile of NK cells, which may influence their therapeutic impact.

The functionality of anti‐Hsp70 CAR NK cells was validated by co‐culturing with target cells differing in mHsp70 expression. Both anti‐Hsp70 CAR NKL and NK92 cells showed robust responses against tumor target cells with high mHsp70 expression, marked by elevated GrB and IFN‐γ secretion. In contrast, low cytokine secretion in response to mHsp70‐negative THP‐1 cells confirmed antigen dependency and specificity of the CAR. Importantly, CAR‐transduced NK cells exhibited superior killing efficiency compared with Unt NK cells, demonstrating their enhanced capacity for tumor cell elimination and underscoring the impact of CAR engineering in potentiating NK cell‐mediated lysis. The specificity of this cytotoxic response was further validated using anti‐Hsp70 CAR KHYG1 and YT cells, which are prone to CAR loss over time, likely due to exhaustion‐associated downregulation and fratricide driven by a CAR‐induced mHsp70 expression. The absence of enhanced killing in these cell lines confirms that the observed antitumor effects were attributable to specific recognition of mHsp70 on target cells, rather than intrinsic NK cell line variations. These observations underscore the robustness and antigen‐specific functionality of CAR NK cells across diverse NK cell platforms. Tumor cells that survived anti‐Hsp70 CAR NK cell treatment exhibited a lower surface mHsp70 expression. This finding indicates a potential immune‐evasion mechanism whereby tumor cells may downregulate mHsp70 to escape immune surveillance.

Importantly, while NK cell lines such as NK92 provide robust and reproducible models for mechanistic studies and preclinical CAR NK cell development, their malignant origin and adaptation to artificial culture conditions limit their direct clinical translation [75]. Although NK92 cells have been used in clinical trials, they require irradiation before infusion to prevent engraftment, which reduces persistence and efficacy [76]. In contrast, primary NK cells, with their superior safety profile and physiological function, or clinical‐grade, GMP‐compliant CAR NK cell products derived from allogeneic NK cells or induced pluripotent stem cells (iPSCs), offer more promising platforms for clinical application [77, 78, 79]. Therefore, the present study should be viewed as a proof of concept for Hsp70‐targeting CAR NK strategies, setting the stage for translation and optimization in primary and iPSC‐derived NK cell platforms. Such a transition will be essential to assess the clinical potential of anti‐Hsp70 CAR NK therapies. Additionally, while our data demonstrate that CAR engineering enhances NK cell activation and cytotoxicity, we also observed that parental NK cells exhibited cytotoxic responses against mHsp70‐positive tumor cells, which our previous studies suggest may be mediated through CD94‐dependent recognition of mHsp70 [39, 52]. Given the consistent upregulation of CD94 following TKD/IL‐2 priming and CAR transduction, it is plausible that CD94 not only facilitates the natural cytotoxicity of Unt NK cells but may also act as a co‐stimulatory molecule amplifying CAR‐mediated activation against mHsp70‐positive targets. Although the present study did not directly dissect this mechanism, future experiments employing CD94 blockade or knockdown will clarify its contribution to CAR NK cell cytotoxicity.

## 4 Conclusion

In summary, we established a novel CAR NK cell platform targeting mHsp70, a tumor‐specific antigen broadly expressed on highly aggressive solid tumors, including HNSCC, and our study highlights the importance of selecting a clinically relevant antigen and a compatible NK cell platform for optimal CAR‐based therapy. The selective and potent cytotoxicity of these engineered NK cells, coupled with their immunological specificity, makes this approach promising for cancer immunotherapy. Notably, the exclusive expression of mHsp70 on tumor cells but not on normal tissues, and its widespread presence across various tumors, further support the specificity, safety, and utility of this strategy, reducing off‐target effects. The interaction between the scFv domain of the anti‐Hsp70 CAR and the binding domain of mHsp70 was confirmed through in silico modeling and validated by in vitro assays, demonstrating that the CAR–mHsp70 interaction is structurally feasible and functionally effective. CAR‐engineered NK cells exhibited strong cytotoxicity and cytokine release against mHsp70‐positive tumor cells, whereas mHsp70‐negative targets and Unt NK cells showed minimal responses, confirming antigen‐specific activity. The stronger activation and cytotoxic responses observed in CAR NK cells compared with naïve NK cells following TKD/IL‐2 stimulation further support the mechanistic linkage between mHsp70 recognition and NK cell activation. However, the emergence of tumor populations with low mHsp70 expression after treatment stresses a potential mechanism of immune evasion and indicates the need to develop complementary strategies to overcome antigen loss and maintain long‐term tumor control. Future studies using in vivo models and combination approaches [80], including assessments of bystander killing, will be essential to comprehensively evaluate the durability, safety, and clinical relevance of anti‐Hsp70 CAR NK cells. Collectively, these findings provide valuable insights in advancing CAR NK cell therapies and strengthening the role of mHsp70 as a promising, specific, and broad‐spectrum target for treating aggressive tumors.

## 5 Materials and Methods

Detailed protocols for protein labeling, confocal microscopy, flow cytometric quantification of mHsp70, computational modeling and simulation, microscale thermophoresis, NK cell immunophenotyping, NK cell expansion, and functional readout assays are provided in the Supplementary Information together with reagent specifications and extended methodological descriptions.

### 5.1 Cell Lines and Cell Culture

Cal27, SAS, and UD‐SCC‐5 cells were cultured in high‐glucose Dulbecco's modified Eagle medium (DMEM), supplemented with 10% heat‐inactivated fetal bovine serum (FBS). THP‐1 cells were maintained in Roswell Park Memorial Institute medium (RPMI)‐1640 containing 10% FBS. NK cell lines (YT, KHYG1, NKL, and NK92) and their anti‐Hsp70 CAR‐engineered counterparts were cultured in RPMI‐1640 supplemented with 20% FBS and 1% non‐essential amino acids. Cells were maintained under standard culture conditions and routinely tested for mycoplasma contamination.

### 5.2 Generation of Anti‐Hsp70 CAR NK Cells

The human anti‐Hsp70 CAR construct (Hsp70p‐CD28‐CD3ζ), incorporating the cmHsp70.1 mAb‐derived scFv into a CD28‐CD3ζ backbone, was introduced into NK cell lines by RetroNectin‐assisted retroviral transduction using pMP71 vectors and established packaging cell lines, as previously described [81]. Transduced NK cells were expanded in IL‐2‐containing medium and harvested for downstream analyses. CAR expression was assessed by flow cytometry using anti‐c‐Myc mAb and Hsp70 protein, and CAR‐positive NK cells were enriched by flow cytometric sorting based on dual positivity for c‐Myc and Hsp70 binding.

### 5.3 Functional Analysis of NK Cell Activity

Soluble cytokines and cytotoxic mediators in cell culture supernatants were quantified using MACSPlex Cytokine 12 and Cytotoxic T/NK Cell kits following NK cell priming with TKD and IL‐2. Secretion of IFN‐γ and GrB after target cell exposure was further analyzed by dual‐color FluoroSpot assay at an effector‐to‐target (E:T) ratio of 1:1. NK cell‐tumor cell interactions and target cell killing were monitored by live cell time‐lapse imaging using fluorescently labelled effector and target cells together with SYTOX Blue staining for dead‐cell identification. Target cell death was additionally quantified by Annexin V‐FITC/PI staining after co‐culture with NK cells, with an anti‐CD45 antibody used to distinguish NK cells from HNSCC cells.

### 5.4 Statistical Analysis

Statistical analyses were performed using GraphPad Prism (version 10.1.2). Data distribution was assessed using the Shapiro–Wilk test, followed by parametric or non‐parametric tests depending on data normality and group number, including Student's *t*‐test, Mann–Whitney *U* test, Kruskal–Wallis test, or two‐way ANOVA with post hoc analysis. Data are presented as mean ± standard deviation (SD). Statistical significance was defined as *p* ≤ 0.05.

## Author Contributions

K.H. performed experiments, analyzed data, prepared figures, and contributed to drafting the manuscript. M.Y. and A.B.D. analyzed data, prepared figures, and contributed to manuscript drafting and review. F.K.M. and M.D.S. carried out the in silico analyses. C.C. and M.P.T. generated the CAR‐engineered cells. A.T., M.H.K., C.C.H., A.G., B.A., and J.A. contributed to methodology development and performed experiments. M.Y., J.A., A.G.P., E.W., B.W., and S.K. reviewed and edited the manuscript. G.M. and A.B.D. were responsible for conceptualization, supervision, funding acquisition, and final manuscript review. All authors have read and approved the final version of the manuscript.

## Funding

G.M. and A.B.D. received funding from BAYCELLator (AZ‐1568‐22). G.M. was supported by a grant from the BMBF (02NUK064 A/B). B.A. was supported by a BMFTR grant (02NUK064B). S.K. is supported by the Bavarian Cancer Research Center (BZKF) (TANGO), the Deutsche Forschungsgemeinschaft (DFG, grant number: KO5055‐2‐1 and KO5055/3‐1 to S.K.;), the international doctoral program “i‐Target: immunotargeting of cancer” (funded by the Elite Network of Bavaria), the Melanoma Research Alliance (grant number 409510), Marie Sklodowska‐Curie Training Network for Optimizing Adoptive T Cell Therapy of Cancer (funded by the Horizon 2020 programme of the European Union; grant 955575), Marie Sklodowska‐Curie Training Network for tracking and controlling therapeutic immune cells in cancer (funded by the Horizon Programme of The EU, grant 101168810), Else Kröner‐Fresenius‐Stiftung (IOLIN), German Cancer Aid (AvantCAR.de), the Wilhelm‐Sander‐Stiftung, Ernst Jung Stiftung, Institutional Strategy LMUexcellent of LMU Munich (within the framework of the German Excellence Initiative;), the Go‐Bio‐Initiative, the m4‐Award of the Bavarian Ministry for Economical Affairs, Bundesministerium für Bildung und Forschung, the EUROSTAR‐Programm, European Research Council (Starting Grant 756017, PoC Grant 101100460 and CoG 101124203), by the SFB‐TRR 338/1 2021–452881907, Fritz‐Bender Foundation, Deutsche José Carreras Leukämie Stiftung, Hector Foundation, Bavarian Research Foundation (BAYCELLATOR), the Monika‐Kutzner Foundation, the Bruno and Helene Jöster Foundation (360°CAR), the Dr. Rurainski‐Foundation.

## Ethics Statement

All experiments involving NK cell lines were conducted in accordance with institutional biosafety regulations.

## Conflicts of Interest

Gabriele Multhoff is the Founder and Chief Scientific Officer of multimmune GmbH. A. Graham Pockley is the Chief Executive Officer of multimmune GmbH. Sebastian Kobold has received honoraria from Plectonic, TCR2 Inc., Miltenyi, Galapagos, Cymab, Novartis, BMS, and GSK, is an inventor of several patents in the field of immuno‐oncology, received license fees from TCR2 Inc and Carina Biotech, and received research support from TCR2 Inc., Tabby Therapeutics, Catalym GmbH, Plectonic GmbH, and Arcus Bioscience for work unrelated to the manuscript. The remaining authors declare no conflicts of interest.

## Supporting information




**Figure S1**: Representative example of gating strategy for flow cytometric analysis of membrane‐bound Hsp70 (mHsp70) expression by cancer cell lines. (A) The cancer cell population was identified based on side/forward scatter properties, (B) single cell characteristics, (C) viability (PI negative), and (D) mHsp70 expression based on the cell surface binding of FITC‐cmHsp70.1 monoclonal antibody (mAb) (green histogram). Isotype‐matched controls in gray. The percentage of mHsp70‐positive cells and the fold change in mean fluorescence intensity (MFI) were determined relative to the isotype‐matched control.
**Figure S2**: Comparison of the amino acid sequences of light and heavy chains between Hsp70 and modelled antibodies using the BLAST sequence analysis tool. (A) Summary table of sequence alignments. (B) Alignment results of the light chain (chain L) from the antibody structure (PDB ID: 7U62) and the heavy chain (chain A) from PDB ID: 7OO2, aligned against light and heavy chain sequences of the mHsp70 mAb using CLC Sequence Viewer.
**Figure S3**: *Apo* single‐chain variable fragment (scFv) conformational clustering and structural modeling workflow. A) Structural modeling pipeline showing individual light and heavy chain templates (PDB: 7oo2 and 7u62), which were docked to generate the top *apo* scFv model (docking score: −338.78 kcal/mol), followed by three independent 150 ns molecular dynamics (MD) simulations and clustering of resulting conformations into three representative clusters. (B) RMSD matrix comparing intercluster backbone deviations among three dominant *apo* (backbone) scFv conformational clusters, illustrating structural heterogeneity. (C) Hierarchical clustering dendrogram of MD frames based on backbone RMSD (in units of Å), showing three distinct conformational populations. (D) Bar graph showing the population distribution within each cluster.
**Figure S4**: Conformational clustering and structural modeling workflow of the *holo* scFv‐TKD peptide complex. (A) Docking scores (in kcal/mol) of the TKD peptide to three representative *apo* scFv conformations, calculated using the HDOCK server. (B) Root‐mean‐square deviation (RMSD) matrix (in Å) comparing backbone conformational differences among the three *holo* scFv clusters, highlighting structural divergence. (C) Hierarchical clustering dendrogram of *holo* scFv conformations derived from MD trajectories, revealing three dominant structural populations. (D) Distribution of cluster populations based on the number of trajectory frames assigned to each conformational state. (E) Structural snapshots of the scFv–TKD complex in the top‐docked pose and after 150 ns MD simulations for each cluster, with magnified views of conformational differences in the CDR‐H3 of the antibody.
**Figure S5**: Structural stability and flexibility analysis of the scFv–TKD complex. (A) RMSD values (nm); (B) root mean square fluctuation (RMSF) values (nm).
**Figure S6**: Binding affinity of the cmHsp70.1 mAb to recombinant Hsp70 protein measured by Microscale Thermophoresis (MST). (A) MST traces depict the thermophoretic response of cmHsp70.1 mAb across a gradient of Hsp70 protein concentrations. The blue and red shaded regions represent the cold and hot measurement intervals, respectively, from which normalized fluorescence (Fnorm, ‰) was calculated. (B) Capillary fluorescence scans reveal uniform fluorescence signals across all loaded capillaries, confirming the absence of surface adsorption, protein aggregation, or ligand‐induced fluorescence artifacts. (C) The dose–response curve describes the interaction between FITC‐labeled cmHsp70.1 and serial dilutions of full‐length recombinant Hsp70, with Fnorm plotted against ligand concentration (M). Nonlinear curve fitting yielded a dissociation constant (KD = 1.91 nM), indicating high‐affinity binding.
**Figure S7**: Representative example of the gating strategy for flow cytometric analysis of CD94 and CD69 expression on untransduced (Unt) and anti‐Hsp70 CAR‐transduced NKL cells. (A) The NKL cell population was identified by side/forward scatter properties, (B) single cell characteristics, (C) viability (SYTOX Blue negative), and (D) the cell surface markers CD94 or CD69 using specific fluorescently conjugated mAbs. The percentage of positively stained cells and MFI fold change were determined relative to isotype‐matched controls.
**Figure S8**: mHsp70 expression on the surface of THP‐1 cells. (A) Representative confocal microscopy images of THP‐1 cells (negative control) stained with FITC‐cmHsp70.1 mAb to detect mHsp70 expression (green). Nuclei were counterstained with Hoechst 33342 (blue), and the filamentous actin (F‐actin) cytoskeleton was visualized using Alexa Fluor Plus 647 Phalloidin (red). The scale bar represents 50 µm. (B) Representative flow cytometric measurement of mHsp70 expression in the THP‐1 cell line using FITC‐cmHsp70.1 mAb. Green histograms indicate mHsp70 expression, while gray histograms correspond to isotype‐matched controls (mouse FITC‐IgG1 mAb). The mHsp70 expression is calculated as the percentage of mHsp70‐positive viable cancer cells.
**Figure S9**: Representative ELISpot images of secreted Granzyme B (GrB) and interferon‐gamma (IFN‐γ) by NK cells after co‐culture with mHsp70‐positive and negative tumor cells. The CAR‐transduced and Unt NK cells were co‐cultured with THP‐1 or Cal27 cells at an E:T ratio of 1:1 for 6 h. Following the dual‐color FluoroSpot assay, spots representing individual cells that have secreted GrB (red) and IFN‐γ (green) were counted using an ImmunoSpot analyzer. The total numbers of spots per well are indicated in the upper‐right corner of each image.
**Figure S10**: Representative time‐lapse fluorescence microscopy to assess NK cell‐mediated cytotoxicity against target cancer cells. Unt NKL (left) and NK92 (right) cells were co‐incubated with HNSCC target cells (Cal27, UD‐5, SAS) at an effector‐to‐target (E:T) ratio of 1:1. The THP‐1 cells served as control cells. Target cells were pre‐labeled with PKH67 (green), and SYTOX Blue was added to detect dead cells (blue), thereby enabling visualization of NK cell‐mediated cytotoxic effects over time. Monitoring was performed for 20 h, and images captured at 1, 6, and 12 h after co‐incubation are shown representatively. The labelled NK cells were excluded from the images to allow better visualization of the dynamic changes in the cancer cells. The scale bar is 100 µm.
**Figure S11**: Flow cytometric analysis of apoptotic and necrotic cells stained with Annexin V‐FITC and PI. Cal27, UD‐5, and SAS target cells (2 × 10^5^ cells per well) with high Hsp70 expression and THP‐1 cells with low or no mHsp70 expression were co‐cultured with (A) YT and (B) KHYG1 (either CAR‐transduced or Unt cells) effector NK cells at 37°C for 6 h at an E:T ratio of 1:1 before Annexin V/PI staining and flow cytometry analysis (mean ± SD, *n* ≥ 3 independent experiments).
**Figure S12**: Representative example of gating strategy for flow cytometry analysis of apoptosis and necrosis in target cell lines using an Annexin V/PI assay after a 6‐h co‐incubation with NK effector cells. A) Effector and target cells were gated based on side/forward scatter properties, (B) single‐cell characteristics, (C) the leukocyte marker CD45, and (D) Annexin V‐FITC vs. PI. Early and late apoptosis, as well as necrosis in the target cancer cell population (CD45^−^), were identified based on the following staining patterns: viable cells (Annexin V^−^/PI^−^), early apoptosis (Annexin V^+^/PI^−^), late apoptosis (Annexin V^+^/ PI^+^), and necrosis (Annexin V^−^/PI^+^).
**Figure S13**: Schematic representation of the experimental workflow used for the generation and functional assessment of anti‐Hsp70 CAR‐engineered NK cells. A second‐generation CAR construct targeting mHsp70 was packaged into retroviral particles using HEK 293T cells and transduced into NK cell lines (YT, KHYG1, NKL, and NK92). The transduced CAR NK cells were expanded and stimulated with Hsp70‐derived TKD peptide and low‐dose IL‐2, after which their cytotoxic responses against mHsp70‐positive HNSCC cell lines were assessed. Functional assays included measurement of effector molecules (e.g., IFN‐γ, granzyme B, perforin), Annexin V/PI‐based cytotoxicity analysis, and evaluation of antigen specificity using mHsp70‐negative cells. Structural insights into CAR‐antigen interactions were further supported by molecular docking and molecular dynamics simulation analyses. This figure was created with BioRender.com.

## Data Availability

Data will be made available from the corresponding author upon reasonable request.
