# Supplementary material for: Development of CAR NK Cell Lines Selectively Targeting Cancer Cells Expressing Membrane Hsp70
Source: MedComm (2020). 2026 May 10;7(5):e70753. doi: 10.1002/mco2.70753 (PMC13158374; doi:10.1002/mco2.70753)
Supplement: Supplementary file 1 — Figure S1: Representative example of gating strategy for flow cytometric analysis of membrane‐bound Hsp70 (mHsp70) expression by cancer cell lines. (A) The cancer cell population was identified based on side/forward scatter properties, (B) single cell characteristics, (C) viability (PI negative), and (D) mHsp70 expression based on the cell surface binding of FITC‐cmHsp70.1 monoclonal antibody (mAb) (green histogram). Isotype‐matched controls in gray. The percentage of mHsp70‐positive cells and the fold change in mean fluorescence intensity (MFI) were determined relative to the isotype‐matched control. Figure S2: Comparison of the amino acid sequences of light and heavy chains between Hsp70 and modelled antibodies using the BLAST sequence analysis tool. (A) Summary table of sequence alignments. (B) Alignment results of the light chain (chain L) from the antibody structure (PDB ID: 7U62) and the heavy chain (chain A) from PDB ID: 7OO2, aligned against light and heavy chain sequences of the mHsp70 mAb using CLC Sequence Viewer. Figure S3: Apo single‐chain variable fragment (scFv) conformational clustering and structural modeling workflow. A) Structural modeling pipeline showing individual light and heavy chain templates (PDB: 7oo2 and 7u62), which were docked to generate the top apo scFv model (docking score: −338.78 kcal/mol), followed by three independent 150 ns molecular dynamics (MD) simulations and clustering of resulting conformations into three representative clusters. (B) RMSD matrix comparing intercluster backbone deviations among three dominant apo (backbone) scFv conformational clusters, illustrating structural heterogeneity. (C) Hierarchical clustering dendrogram of MD frames based on backbone RMSD (in units of Å), showing three distinct conformational populations. (D) Bar graph showing the population distribution within each cluster. Figure S4: Conformational clustering and structural modeling workflow of the holo scFv‐TKD peptide complex. (A) Docking scor [file MCO2-7-e70753-s001.pdf]

Development of CAR NK Cell Lines Selectively Targeting Cancer Cells Expressing Membrane Hsp70

Khouloud Hachani[1], Mina Yazdi[2,*], Charlotte Carcopino[3], Fatemeh Khademi Moghadam[4], Micholas Dean Smith[4], Anskar Trill[1,5], Marcel P. Trefny[3], Morteza Hasanzadeh Kafshgari[5,6], Cosima C. Hoch[1], Abdallah Gaballa[7,8,9], Bayan Alkotub[5,10,11], Jennifer Altomonte[12], A. Graham Pockley[13], Ernst Wagner[2], Barbara Wollenberg[1], Sebastian Kobold[3,14,15,16], Gabriele Multhoff[5,†], Ali Bashiri Dezfouli[1,5,*,†]

[1] Department of Otolaryngology, Head and Neck Surgery, TUM School of Medicine and Health, Technical University of Munich, Munich, Germany

[2] Pharmaceutical Biotechnology, Department of Pharmacy, Ludwig-Maximilians-Universität (LMU), Munich, Germany

[3] Division of Clinical Pharmacology, Department of Medicine IV, University Hospital, Ludwig-Maximilians-Universität (LMU), Munich, Germany

[4] Department of Biochemistry and Cellular and Molecular Biology, The University of Tennessee, Knoxville, TN, USA

[5] Central Institute for Translational Cancer Research (TranslaTUM), TUM School of Medicine and Health, Technical University of Munich, Munich, Germany

[6] Heinz-Nixdorf-Chair of Biomedical Electronics, Campus Klinikum München rechts der Isar, TranslaTUM, Technical University of Munich, Munich, Germany

[7] Division of Translational Cancer Research, German Cancer Research Center and German Cancer Consortium, Heidelberg, Germany

[8] Chair of Translational Cancer Research and Institute of Experimental Cancer Therapy, Klinikum Rechts der Isar, School of Medicine, Technical University of Munich, Munich, Germany.

[9] Center for Translational Cancer Research (TranslaTUM), School of Medicine, Technical University of Munich, Munich, Germany.

[10] Institute of Biological and Medical Imaging, Bioengineering Center, Helmholtz Zentrum München, Neuherberg, Germany

[11] Chair of Biological Imaging, Central Institute for Translational Cancer Research (TranslaTUM), School of Medicine and Health, Technical University of Munich, Munich, Germany

[12] Department of Internal Medicine II, Klinikum Rechts der Isar, Technical University of Munich, Munich, Germany

[13] multimmune GmbH, 81675 Munich, Germany

[14] German Cancer Consortium (DKTK), a partnership between LMU University Hospital and DKFZ, Heidelberg, Germany

[15] German Center for Lung Research (DZL), partner site Munich, Germany

[16] Einheit für Klinische Pharmakologie (EKLiP), Helmholtz Zentrum München - German Research Center for Environmental Health Neuherberg, Germany

***Correspondence:**
M. Yazdi, A. Bashiri Dezfouli
mina.yazdi@cup.uni-muenchen.de; ali.bashiri@tum.de

[†] Equally contributed as senior authors.

Materials and Methods

1. Cell Lines and Cell Culture

Cal27 and SAS cells, originating from human oral squamous cell carcinoma, and UD-SCC-5 cells, derived from laryngeal squamous cell carcinoma, were generously provided by Prof. Dr. Barbara Wollenberg. The cells were cultured in Dulbecco's Modified Eagle Medium (DMEM) containing high glucose (4.5 g/L; Sigma-Aldrich), supplemented with 10% v/v heat-inactivated fetal bovine serum (FBS; Sigma-Aldrich). THP-1 cells, a human monocytic leukemia cell line, were grown in Roswell Park Memorial Institute Medium (RPMI)-1640 (Sigma-Aldrich) supplemented with 10% v/v FBS. The NK cell lines (YT, KHYG1, NKL, and NK92) and their genetically engineered counterparts containing the anti-Hsp70 CAR were cultured in RPMI-1640 supplemented with 20% v/v FBS and 1% v/v non-essential amino acids (Sigma-Aldrich). All cell culture media contained 1% v/v antibiotics (100 IU/mL penicillin and 100 µg/mL streptomycin; Sigma-Aldrich), 2 mM L-glutamine (Sigma-Aldrich), and 1 mM sodium pyruvate (Sigma-Aldrich). All cells were maintained at 37 °C, 95% v/v humidity and 5% v/v CO_2 atmosphere and were routinely tested for mycoplasma contamination. Cell viability was tested before each experiment using Trypan blue dye exclusion.

2. Protein Labeling and Fluorescent Tracing

Recombinant Hsp70 protein (multimmune GmbH), bovine serum albumin (BSA; Sigma-Aldrich), and the cmHsp70.1 mAb (multimmune GmbH) were labeled following a standardized protocol which has been described previously [1]. Briefly, carbonate buffer (1 M) was added to each protein solution (1 mg/mL in double-distilled water) in a 1:10 v/v ratio, followed by the addition of 50 µL of FITC (10 mg/mL in 0.1 M carbonate buffer; Sigma-Aldrich). The mixture was incubated overnight in the dark at 4 °C with gentle shaking. After incubation, the labeled proteins were dialyzed using Slide-A-Lyzer™ Dialysis Cassettes (ThermoFisher Scientific). The protein concentration and dye-to-protein ratio were assessed using a microplate spectrophotometer (PerkinElmer). To prevent bacterial contamination, 0.02% w/v sodium azide was added to the final solution, which was stored at 4 °C in the dark. In a separate labeling step, recombinant Hsp70 protein and BSA were labelled for flow cytometry and confocal microscopy using the Alexa Fluor™ 647 Microscale Protein Labeling Kit (ThermoFisher Scientific).

3. Confocal Microscopy

To visualize mHsp70 expression, cancer cells (2×10^4 cells/well) were seeded on poly-L-lysine (Sigma-Aldrich) coated slides (ThermoFisher Scientific). After 24 h, the medium was replaced with a complete medium containing FITC-conjugated cmHsp70.1 mAb for 1 h at 4 °C. Cells were washed twice using ice-

cold flow cytometry buffer composed of phosphate buffered saline (PBS) containing 10% v/v FBS and then fixed with 3.7% w/v paraformaldehyde (PFA) in PBS for 15 min at room temperature (RT), followed by another washing step. Following fixation, cells were permeabilized with buffer (Invitrogen), prepared by diluting the 10X stock with PBS (1:10 v/v), for 1 h at RT. Filamentous actin (F-actin) and nuclei were stained using Alexa Fluor™ Plus 647 Phalloidin (1 µg/mL; ThermoFisher Scientific) for 30 min and Hoechst 33342 dye (5 µg/mL; Sigma-Aldrich) for 10 min at RT under light-protected conditions, respectively. Finally, cells were washed and subjected to microscopy.

The binding of fluorescently labeled Hsp70 to the anti-Hsp70 CAR on NK cells was determined using confocal imaging. For this, NK cells (3×10^4) were incubated with Alexa Fluor™ 647-conjugated Hsp70 protein (50 µg/mL) and FITC-conjugated anti-c-Myc mAb (clone REAL810, Miltenyi Biotec) for 15 min on ice, washed with ice-cold flow cytometry buffer, after which nuclei were counter-stained with Hoechst 33342 (5 µg/mL) for 10 min. Images were taken using a Leica TCS SP8 confocal laser-scanning microscope (CLSM, Germany) using an HC PL APO 63 × 1.4 objective and Leica LAS X software.

4. Quantification of mHsp70 Expression by Flow Cytometry

The expression of mHsp70 on tumor and NK cell lines was measured by flow cytometry using a MACSQuant® Analyzer 10 (Miltenyi Biotec, Germany). Briefly, cells (3×10^5) were incubated with FITC-conjugated cmHsp70.1 mAb (40 µg/mL) in flow cytometry buffer for 30 min on ice in the dark, followed by two washes and resuspension in flow cytometry buffer containing propidium iodide (PI, 10 µg/mL; Invitrogen). For each sample, a minimum of 2×10^4 viable cells were acquired. An isotype-matched immunoglobulin (mouse IgG1 FITC; clone X40, BD Biosciences) served as a control to define the mHsp70-positive gate/population. The mean fluorescence intensity (MFI) ratio was calculated by dividing the MFI of FITC-cmHsp70.1-stained cells by the MFI of the isotype control cells. Quantification of mHsp70 molecules per cell was performed using BD Quantibrite™ PE Phycoerythrin Fluorescence Beads (340495, BD Biosciences) which established a calibration curve based on MFI values. FlowJo™ software (version 10.10) was used for data analysis.

5. Computational Modeling and Simulation Methods

Structure Preparation

The amino acid sequences of the scFv light and heavy chains of the anti-Hsp70 CAR, as derived from the cmHsp70.1 mAb, were determined through experimental sequencing. To identify suitable structural templates, BLAST (Basic Local Alignment Search Tool) was performed using the NCBI platform (https://blast.ncbi.nlm.nih.gov/Blast.cgi). Templates with the highest sequence identity (100% for the light

chain and 88.69% for the heavy chain) and optimal coverage (98% and 100% for light and heavy chain, respectively) were selected: PDB ID 7u62 (chain L) [2] for the light chain and 7oo2 (chain A) [3] for the heavy chain. Sequence alignments were further refined using CLC Sequence Viewer (version 8) to confirm compatibility. Due to the distinct structural origins of the light and heavy chains, each was extracted separately and subsequently reassembled into a complete antibody structure using HDOCK, a web-based protein–protein docking platform. For peptide-protein docking, the Hsp70-derived TKD peptide was extracted from the Hsp70 protein (PDB ID: 6K39).

Molecular Docking

The docking of TKD-antibody was performed after generating conformational ensembles from three independent equilibrium MD replicates. Structural similarity clustering, using TTClust, was then applied to represent the conformational diversity of the *apo* structure (antibody only) [4]. From the resulting clustering of the snapshots from the MD simulations, three cluster centers were used as input receptor structures for TKD-antibody docking using the HDOCK server [5]. The resulting complexes were evaluated and ranked based on docking scores to identify the most favorable TKD-antibody complex, with the top-scoring complex further refined by subsequent MD simulations to assess the stability and dynamics of the antibody-peptide-antibody interaction.

MD Simulation

MD simulations were conducted using the GROMACS 2020.6 [6] simulation package with the Amber19SB force field [7, 8] for proteins and the TIP3P water model [9]. The systems were solvated in TIP3P water and neutralized with a NaCl solution (0.1 M) using CHARMM-GUI [10]. Both *apo* and *holo* (antibody–TKD complex) structures were prepared. Each system was simulated for 150 ns in an NPT ensemble at 310 K and 1 atm. The initial box dimensions were 89 Å × 89 Å × 89 Å for the *apo* system and 105.4 Å × 105.4 Å × 105.4 Å for the *holo* system. Periodic boundary conditions were applied throughout. Long-range electrostatics were treated using the Particle Mesh Ewald (PME) method (as implemented in GROMACS) [11, 12], and hydrogen bond constraints were enforced using the P-LINCS algorithm [13]. Short-range electrostatics and van der Waals interactions were treated with a 0.9 nm cutoff using a force-switching scheme.

Following model construction, each system underwent energy minimization using the steepest descent algorithm until the maximum force dropped below 1000 kJ/mol/nm. This was followed by a 125 ps NVT relaxation using the V-rescale thermostat [14] and a 150 ns production NPT simulation using the V-rescale and C-rescale thermo/barostats [15]. Post-relaxation, three independent 150 ns simulations were conducted

for each system (*apo* and *holo*). As described previously, the initial configurations for the *holo* simulations were derived from clustering and docking analyses of the *apo* simulations.

Post-MD Analyses

Following the MD simulations, several post-MD analyses were conducted to evaluate the structural stability, flexibility, and dynamic behavior of the systems. RMSD and Root Mean Square Fluctuation (RMSF) analyses were employed to assess the conformational stability and residue-level flexibility for both the *apo* and *holo* structures. PCA was conducted to identify the dominant motions and to explore the conformational space sampled during the simulations. Additionally, the secondary structure evolution over time was monitored using the DSSP algorithm, offering insights into structural transitions. Hydrogen bond (H-bond) analysis was performed to identify key interactions which contribute to the stability of the antibody-peptide complex. In particular, the number of H-bonds formed between the TKD peptide and the scFv was quantified throughout the simulation using the gmx H-bonds utility with default cutoffs (donor–acceptor distance ≤ 3.5 Å and angle ≥ 150°, corresponding to ≤ 30° deviation from linearity). To extract representative conformations, structural clustering based on backbone RMSD was conducted. This clustering was performed using the TTClust tool following trajectory alignment with GROMACS. It is important to note that PCA and clustering analyses were performed using only the last 125 ns of each independent simulation run. All post-MD analyses were carried out using integrated tools within GROMACS, Visual Molecular Dynamics (VMD), and BIOVIA Discovery Studio Visualizer 2016 software packages.

6. Microscale Thermophoresis (MST)-Based Affinity Measurements

The binding interaction between the cmHsp70.1 mAb and recombinant Hsp70 protein was quantified using MST [16]. FITC-labeled cmHsp70.1 was prepared at a constant concentration and titrated with a serial dilution of recombinant Hsp70 protein (0.000148–11.8 nM). Following a 10-min incubation at RT, samples were loaded into standard capillaries. MST measurements were performed on a Monolith NT.115 instrument (NanoTemper Technologies GmbH) using standard excitation and IR-laser settings recommended by the manufacturer. Thermophoretic movement was recorded, and binding curves were generated by plotting normalized fluorescence Fnorm (‰) against protein concentration. K_D was determined by non-linear fitting using the manufacturer's analysis software.

7. Generation, Characterization, and Sorting of Anti-Hsp70 CAR NK Cells

The human anti-Hsp70 CAR construct (Hsp70p-CD28-CD3ζ) was engineered from an established framework by incorporating a scFv of the cmHsp70.1 mAb that specifically detects mHsp70, into a CD28-CD3ζ backbone. CAR-modification of NK cells was performed using retroviral transduction adapted from

a previously established protocol [17]. For retrovirus production, 293Vec-Galv and 293Vec-RD114 packaging cell lines were used. The retroviral pMP71 vectors carrying the sequence of the relevant CAR receptor were stably introduced into the packaging cells, and single-cell clones were generated and screened indirectly for virus production by assessing transduction efficiency in NK cells. This method was used to generate the 293Vec-RD114 producer cell line for the CAR construct. Viral supernatant containing the retroviral pMP71 vector was collected when the producer cells reached approximately 80% confluency, centrifuged (5 min, 500×g, RT), and passed through Millex-HV 0.45 μm filters (Millipore, Schwalbach, Germany) to remove residual cell debris. One day prior to NK-cell transduction, a non-tissue-culture-treated 24-well plate was coated with 400 µL per well of RetroNectin (1:80 dilution; Takara Bio, Otsu, Japan) in PBS and incubated overnight at 4 °C. The following day, RetroNectin was removed, and the plate was blocked with 500 µL of 2% w/v BSA in PBS for 30 min at RT, washed with PBS, and 2 mL of viral supernatant was added per well. The plate was centrifuged at 3,000×g for 90 min at 37 °C to facilitate virus adsorption. The viral inoculum was removed, wells were rinsed with PBS, and 0.5×10^6 NK cells in 1 mL NK cell medium supplemented with IL-2 (160 IU/mL) were added. Cultures were maintained at 37 °C, fed with fresh medium containing IL-2 on days 1 and 2, and CAR-expressing NK cells were harvested on day 4 for downstream assays.

To evaluate the transduction efficiency, CAR-expressing cells were analyzed by flow cytometry using a MACSQuant® Analyzer 10 following incubation of transduced NK cells with FITC-conjugated anti-c-Myc mAb. Additionally, for specificity assessment, NK cells were incubated with Alexa Fluor™ 647-conjugated Hsp70 protein for 15 min at 4 °C. Incubation with labeled BSA at the same concentration was used as a negative control. The data were analyzed using FlowJo™ software. The percentage of CAR-positive cells was determined based on dual positivity for c-Myc and bound Hsp70 protein.

Following a 4- to 6-day post-transduction incubation, anti-Hsp70 CAR-expressing NK cells were enriched by flow cytometric sorting for c-Myc and Hsp70 dual positivity (BD FACSAria™ Fusion, BD Biosciences). SYTOX™ Blue (Invitrogen) staining was used to confirm cell viability.

8. NK Cell Stimulation and Immunophenotyping

Ex vivo activation was performed by culturing NK cell lines (1×10^6 cells/mL; CAR-transduced, Unt) in RPMI-1640 medium containing IL-2 (160 IU/mL; PeproTech) supplemented with Hsp70-derived peptide (TKD, 2 µg/mL; multimmune GmbH). The following fluorescence-labeled mAbs were used for flow cytometry immunophenotyping: VioGreen™-conjugated anti-CD3 (clone REA613), APC-Vio770-conjugated anti-CD56 (clone REA196), APC-conjugated anti-CD69 (clone REA824), APC-conjugated anti-NKG2C (clone REA205), PE-conjugated anti-NKG2D (clone REA797), APC-conjugated anti-NKp30

(clone REA823), PE-Vio 770-conjugated anti-NKp44 (clone REA1163), PE-Vio 770-conjugated anti-NKp46 (clone REA808), PE-conjugated anti-DNAM-1 (clone REA1040), Vio Bright FITC-conjugated anti-NKG2A (clone REA110), FITC-conjugated anti-KIR2DL2/DL3 (clone REA1006), APC-conjugated anti-KIR3DL1 (clone REA1005), FITC-conjugated anti-PD1 (clone REA1165), PE-Vio 770-conjugated anti-CD94 (clone REA113), PE-conjugated anti-NTB-A (clone REA339), PE-Vio 770-conjugated anti-OX40 (clone REA621), FITC-conjugated anti-LAMP-1 (clone REA792), FITC-conjugated anti-CD253 (TRAIL, clone REA1113), and PE-conjugated anti-CD178 (FasL, clone REA1056), all from Miltenyi Biotec, and PE-conjugated anti-CD16 (clone 3G8, BD Biosciences). Surface receptor expression was quantified on viable, SYTOX™ Blue-negative cells using a MACSQuant® Analyzer 10, and data were analyzed using FlowJo™ software. MFI ratio values were calculated relative to the background staining obtained with isotype-matched control immunoglobulins.

9. Ex Vivo Expansion of NK Cells

NK cell lines were seeded at a density of 5×10^5 cells per well in 24-well plates to evaluate their expansion potential and viability. Proliferation rates were measured by trypan blue dye exclusion, counting every three days over a period of 21 days. NK cell cultures were maintained in RPMI-1640 medium supplemented with IL-2 (160 IU/mL).

10. Multiplex Cytokine Profiling

The MACSPlex Cytokine 12 Kit (human) and MACSPlex Cytotoxic T/NK Cell Kit (human) were used to quantify cytokines/inflammatory mediators (GM-CSF, IFN-α, IFN-γ, IL-4, IL-5, IL-6, IL-9, IL-10, IL-12p70, IL-17A, MCP-1, TNF-α) and cytotoxic markers (granzyme B, perforin, IL-21) in cell culture supernatants. Prior to the experiment, NK cells were primed with TKD and IL-2 for three days, after which supernatants were collected for analysis. According to the manufacturer's instruction, supernatants were incubated with antibody-coated MACSPlex beads, followed by treatment with biotinylated detection reagents to form sandwich complexes. Samples were analyzed by flow cytometry, and cytokine concentrations were determined using known analyte concentrations treated under similar conditions.

11. Dual-Color FluoroSpot Assay for Human IFN-γ / GrB Release

To evaluate the secretion of IFN-γ and GrB by NK cells when exposed to specific target cells, a dual-color FluoroSpot assay was performed according to the manufacturer's instruction (CTL Europe GmbH). Pre-coated ELISpot plates were prepared by washing once with PBS. Cell suspension (100 µL) containing effector and target cells at a ratio of 1:1 was added to each well and the cells were co-incubated for 6 h at 37 °C. After incubation, each well was rinsed twice with PBS and twice with 0.05% v/v Tween-PBS

(TPBS). To visualize the spots, 80 µL of anti-human IFN-γ/GrB detection solution was applied to each well, followed by a 2-h incubation at RT. The plates were then washed three times with TPBS and incubated with 100 µL per well of tertiary solution for an additional 1 h. After three final washes with deionized water, the plates were air dried overnight. Spot quantification was conducted using an ImmunoSpot® analyzer equipped with ELISpot analysis software.

12. Live Cell Time-Lapse Imaging

The effector and target cells were distinctly labelled using PKH fluorescent cell linker kits as per the manufacturer's recommendations (Sigma-Aldrich). Briefly, the effector and target cells (1×10^6 cells) were suspended in 1 mL of PKH26 red dye and PKH67 green dye solution (5x10-7 M in diluent C), respectively. The staining was quenched after 5 min by adding 2 mL FBS. Cells were then centrifuged at 400 g for 10 min, re-suspended in complete medium, thoroughly washed, and transferred into sterile tubes.

For co-culture, PKH67 green-labelled target cells were seeded into 96 well plates at a predetermined density and allowed to adhere for 24 h. Subsequently, PKH26 red-labelled effector cells were added to the wells at a 1:1 ratio. Just before initiating the time-lapse imaging, SYTOX™ Blue (5 µM) was added to enable the identification of dead cells. The cell interactions were continuously monitored for 20 h using an ImageXpress® Micro Confocal High-Content Imaging System with a 10x objective under standard culture conditions. Fluorescence intensity was quantified using MetaXpress v6.5 analysis software.

13. Annexin V/PI Cell Death Assay

Cell death was quantified using an Annexin V-FITC/PI Apoptosis Detection Kit following the manufacturer's instructions (ab14085, Abcam). Cancer cells (2×10^5 cells per well) were co-cultured with effector NK cells at a ratio of 1:1 for 6 h. After incubation, cells were harvested and stained with Annexin V-FITC and PI in binding buffer for 5 min at RT. Staining with APC-conjugated anti-CD45 (Life Technologies, clone HI30) was used to distinguish NK cells ($CD45^+$) from HNSCC cells ($CD45^-$).

14. Statistical Analysis

Statistical analysis was performed using GraphPad Prism (Version 10.1.2). Normality of data distribution was assessed using the Shapiro-Wilk test. For normally distributed data, comparisons between groups were made using an unpaired two-tailed Student's t-test with Welch's correction or one-way ANOVA for multiple groups. For non-normally distributed data, the Mann-Whitney U test was used for two-group comparisons, and the Kruskal-Wallis test was applied for analysis involving more than two groups. Where applicable, post hoc comparisons were conducted using Tukey's or Dunn's test. A two-way ANOVA was performed to evaluate group differences and quantify outcomes derived from time-lapse imaging. Data are

presented as mean ± standard deviation (SD). Significance levels were defined as: not significant (ns), *p < 0.05, **p < 0.01, ***p < 0.001, and ****p < 0.0001.

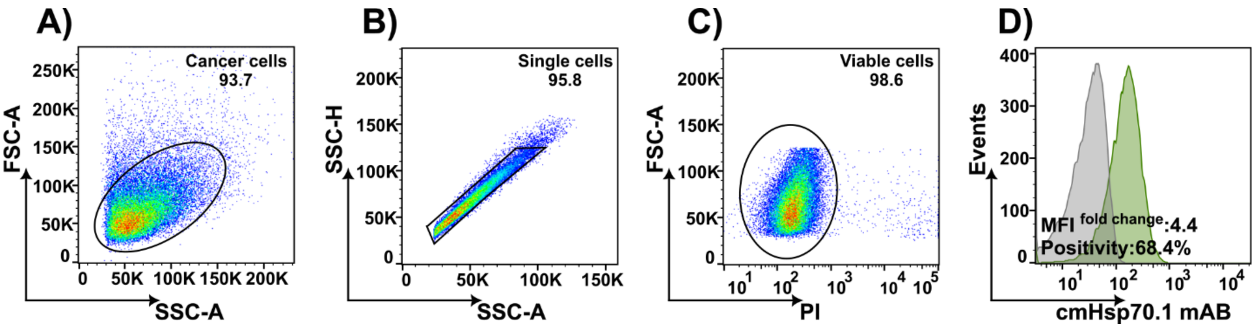

Figure S1. Representative example of gating strategy for flow cytometric analysis of membrane-bound Hsp70 (mHsp70) expression by cancer cell lines. A) The cancer cell population was identified based on side/forward scatter properties, **B)** single cell characteristics, **C)** viability (PI negative), and **D)** mHsp70 expression based on the cell surface binding of FITC-cmHsp70.1 monoclonal antibody (mAb) (green histogram). Isotype-matched controls in gray. The percentage of mHsp70-positive cells and the fold change in mean fluorescence intensity (MFI) were determined relative to the isotype-matched control.

A)

scFv	Description	PDB ID	Query Cover (%)	E value	Identities (%)
Light Chain	Chain L, HY4-1F9 Fab Light Chain [Mus musculus]	7U62	98	3e-157	100.00
Heavy Chain	Chain A, anti-MenX Fab heavy chain [Mus musculus]	7OO2	100	2e-102	88.69

B)

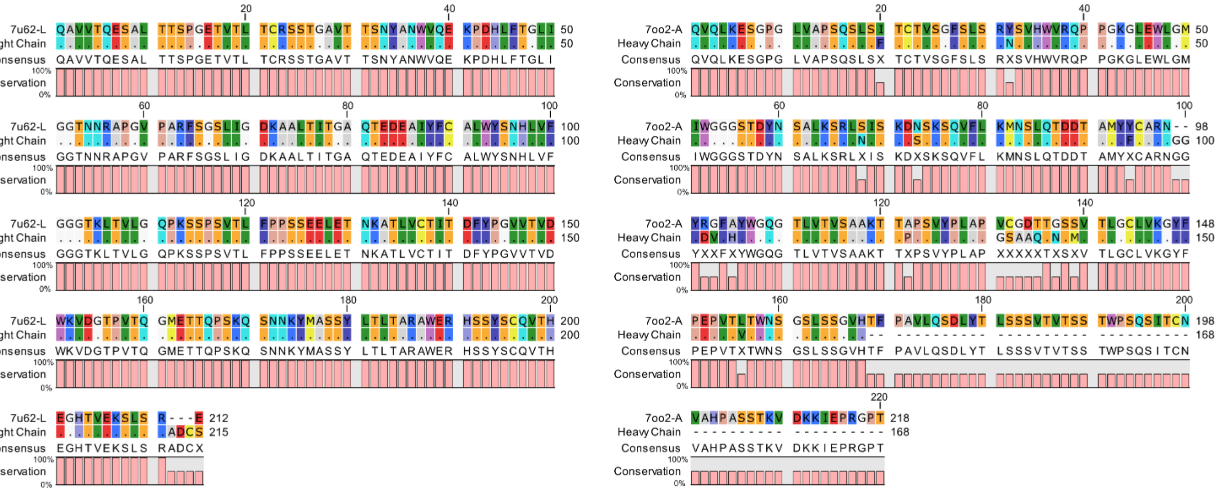

Figure S2. Comparison of the amino acid sequences of light and heavy chains between Hsp70 and modelled antibodies using the BLAST sequence analysis tool. A) Summary table of sequence alignments. **B)** Alignment results of the light chain (chain L) from the antibody structure (PDB ID: 7U62) and the heavy chain (chain A) from PDB ID: 7OO2, aligned against light and heavy chain sequences of the mHsp70 mAb using CLC Sequence Viewer.

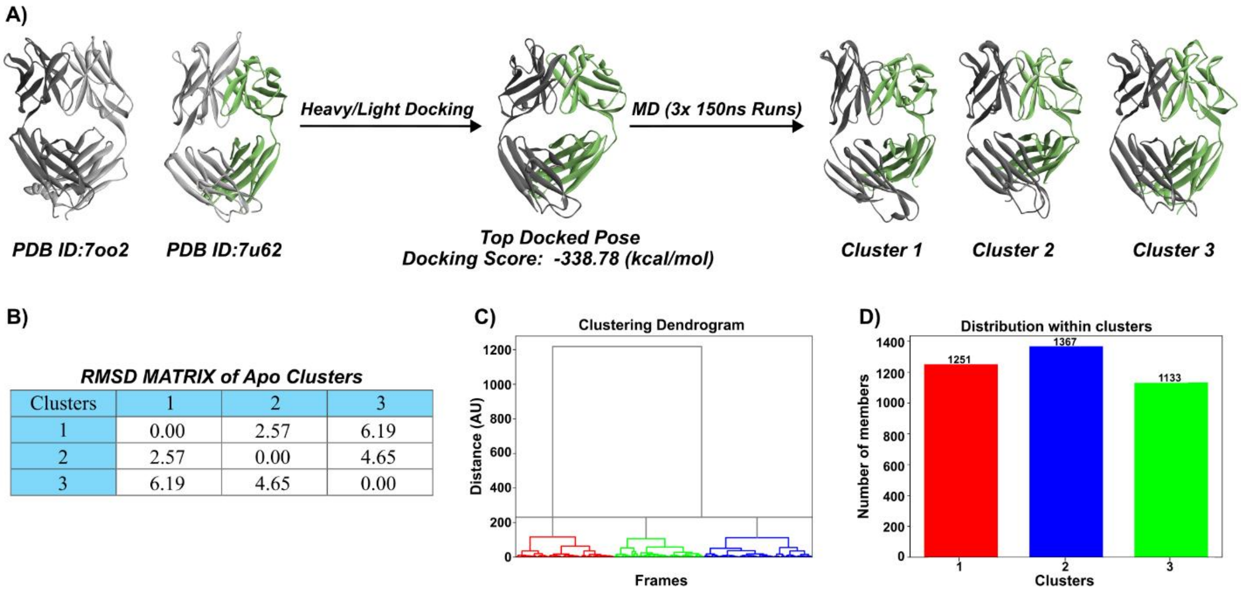

Clusters	1	2	3
1	0.00	2.57	6.19
2	2.57	0.00	4.65
3	6.19	4.65	0.00

Figure S3. *Apo* single-chain variable fragment (scFv) conformational clustering and structural modeling workflow. **A)** Structural modeling pipeline showing individual light and heavy chain templates (PDB: 7oo2 and 7u62), which were docked to generate the top *apo* scFv model (docking score: −338.78 kcal/mol), followed by three independent 150 ns Molecular Dynamics (MD) simulations and clustering of resulting conformations into three representative clusters. **B)** RMSD matrix comparing inter-cluster backbone deviations among three dominant *apo* (backbone) scFv conformational clusters, illustrating structural heterogeneity. **C)** Hierarchical clustering dendrogram of MD frames based on backbone RMSD (in units of Å), showing three distinct conformational populations. **D)** Bar graph showing the population distribution within each cluster.

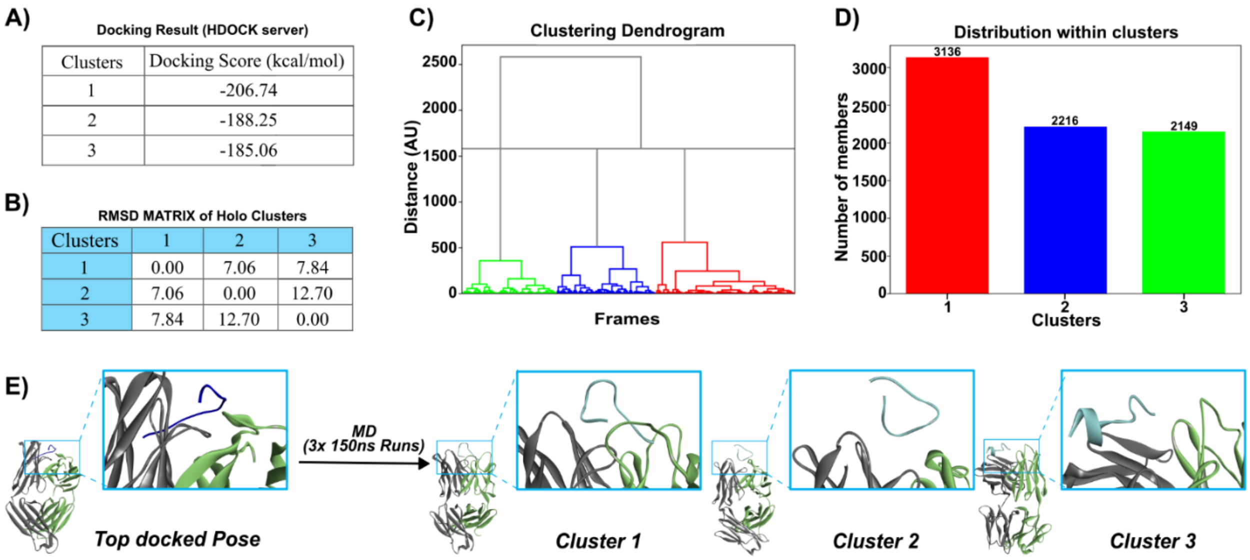

Figure S4. Conformational clustering and structural modeling workflow of the *holo* scFv-TKD peptide complex. **A)** Docking scores (in kcal/mol) of the TKD peptide to three representative *apo* scFv conformations, calculated using the HDOCK server. **B)** Root-mean-square deviation (RMSD) matrix (in Å) comparing backbone conformational differences among the three *holo* scFv clusters, highlighting structural divergence. **C)** Hierarchical clustering dendrogram of *holo* scFv conformations derived from MD trajectories, revealing three dominant structural populations. **D)** Distribution of cluster populations based on the number of trajectory frames assigned to each conformational state. **E)** Structural snapshots of the scFv–TKD complex in the top-docked pose and after 150 ns MD simulations for each cluster, with magnified views of conformational differences in the CDR-H3 of the antibody.

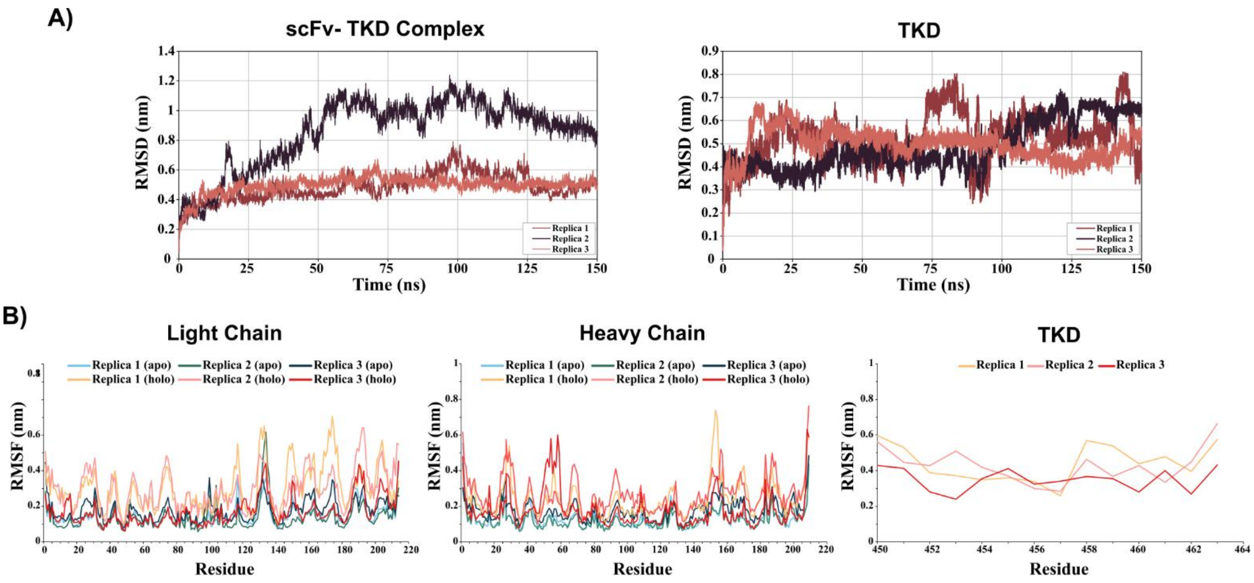

Figure S5. Structural stability and flexibility analysis of the scFv–TKD complex. A) RMSD values (nm), **B)** Root mean square fluctuation (RMSF) values (nm).

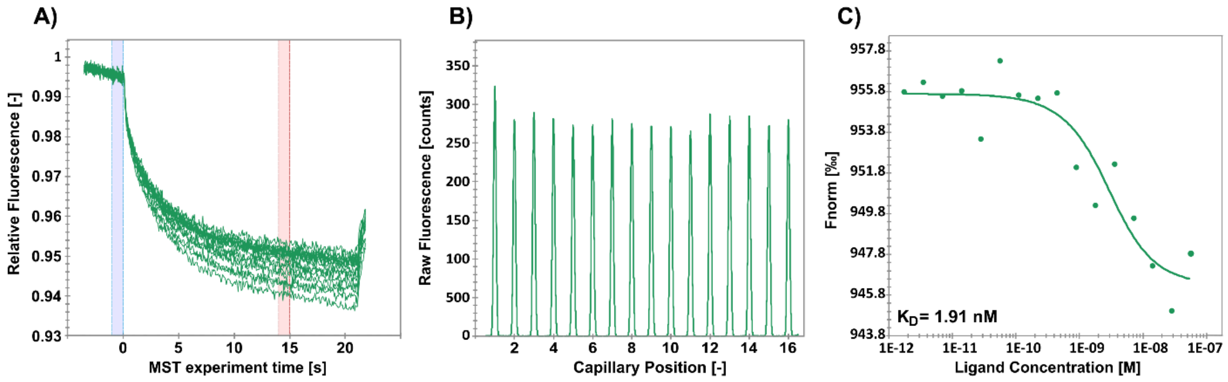

Figure S6. Binding affinity of the cmHsp70.1 mAb to recombinant Hsp70 protein measured by Microscale Thermophoresis (MST). **A)** MST traces depict the thermophoretic response of cmHsp70.1 mAb across a gradient of Hsp70 protein concentrations. The blue and red shaded regions represent the cold and hot measurement intervals, respectively, from which normalized fluorescence (Fnorm, ‰) was calculated. **B)** Capillary fluorescence scans reveal uniform fluorescence signals across all loaded capillaries, confirming the absence of surface adsorption, protein aggregation, or ligand-induced fluorescence artifacts. **C)** The dose–response curve describes the interaction between FITC-labeled cmHsp70.1 and serial dilutions of full-length recombinant Hsp70, with Fnorm plotted against ligand concentration (M). Non-linear curve fitting yielded a dissociation constant (K_D = 1.91 nM), indicating high-affinity binding.

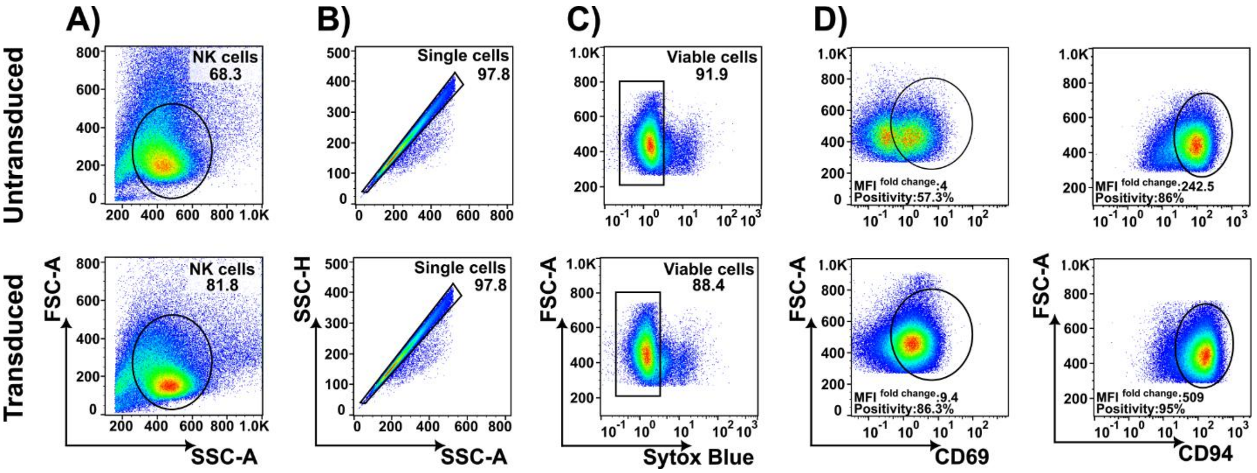

Figure S7. Representative example of the gating strategy for flow cytometric analysis of CD94 and CD69 expression on untransduced (Unt) and anti-Hsp70 CAR-transduced NKL cells. A) The NKL cell population was identified by side/forward scatter properties, **B)** single cell characteristics, **C)** viability (SYTOX™ Blue negative), and **D)** the cell surface markers CD94 or CD69 using specific fluorescently conjugated mAbs. The percentage of positively stained cells and MFI fold change were determined relative to isotype-matched controls.

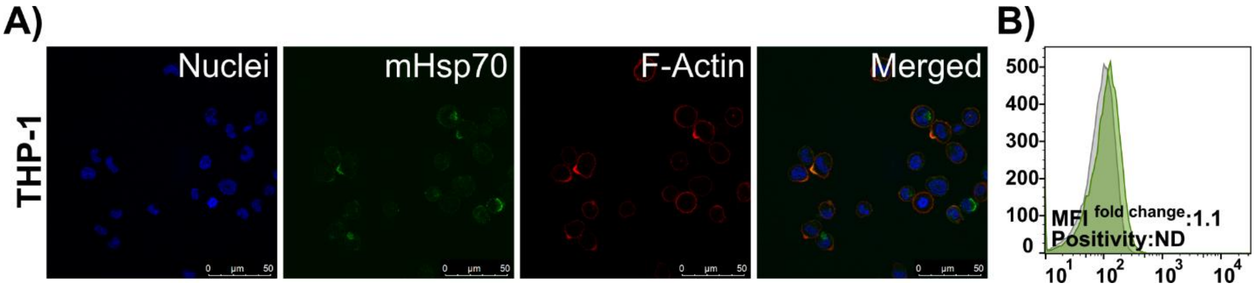

Figure S8. mHsp70 expression on the surface of THP-1 cells. **A)** Representative confocal microscopy images of THP-1 cells (negative control) stained with FITC-cmHsp70.1 mAb to detect mHsp70 expression (green). Nuclei were counterstained with Hoechst 33342 (blue), and the filamentous actin (F-actin) cytoskeleton was visualized using Alexa Fluor™ Plus 647 Phalloidin (red). The scale bar represents 50 µm. **B)** Representative flow cytometric measurement of mHsp70 expression in the THP-1 cell line using FITC-cmHsp70.1 mAb. Green histograms indicate mHsp70 expression, while gray histograms correspond to isotype-matched controls (mouse FITC-IgG1 mAb). The mHsp70 expression is calculated as the percentage of mHsp70-positive viable cancer cells.

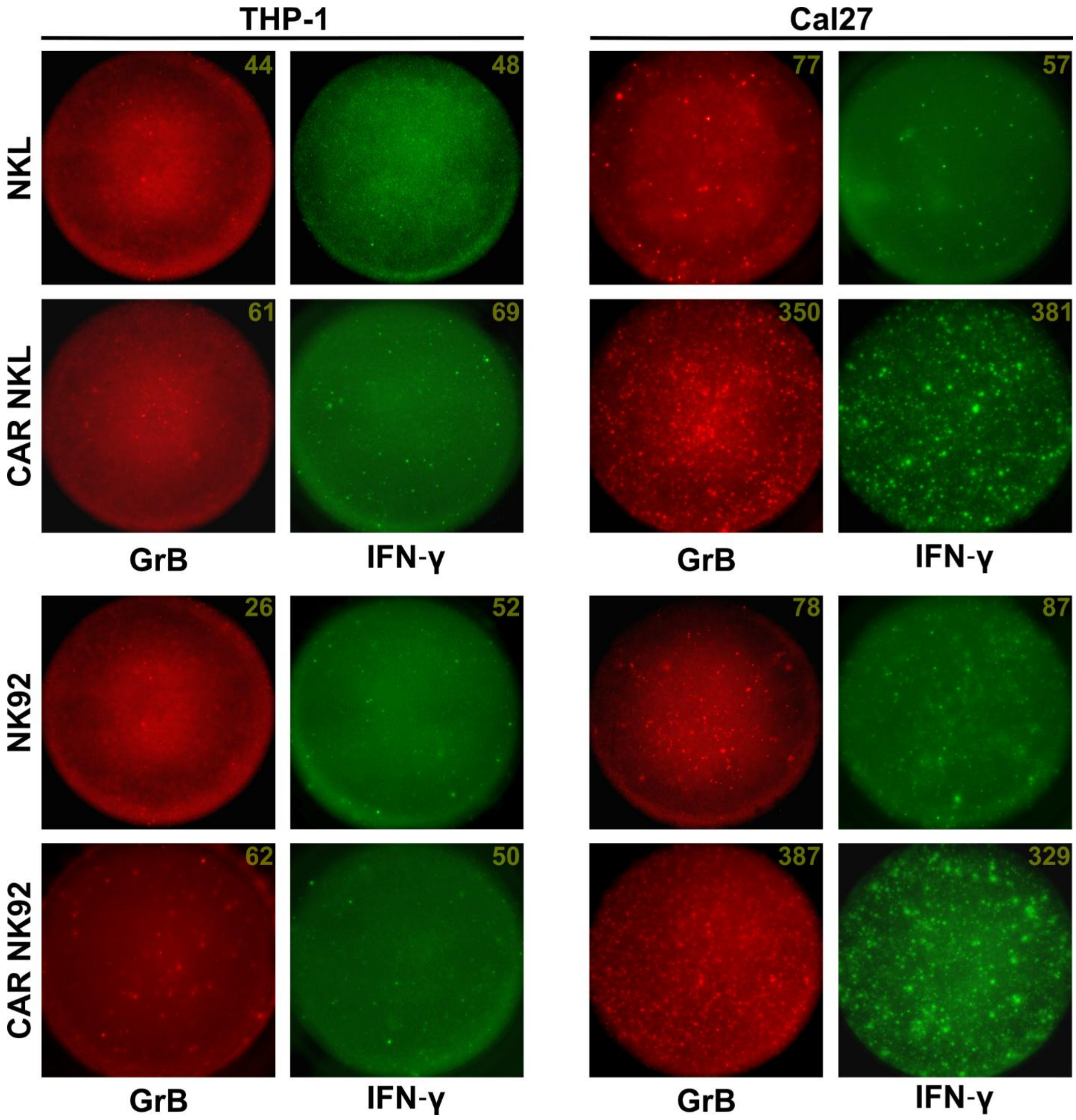

Figure S9. Representative ELISpot images of secreted Granzyme B (GrB) and interferon-gamma (IFN-γ) by NK cells after co-culture with mHsp70-positive and negative tumor cells. The CAR-transduced and Unt NK cells were co-cultured with THP-1 or Cal27 cells at an E:T ratio of 1:1 for 6 h. Following the Dual-color FluoroSpot Assay, spots representing individual cells that have secreted GrB (red) and IFN-γ (green) were counted using an ImmunoSpot analyzer. The total numbers of spots per well are indicated in the upper right corner of each image.

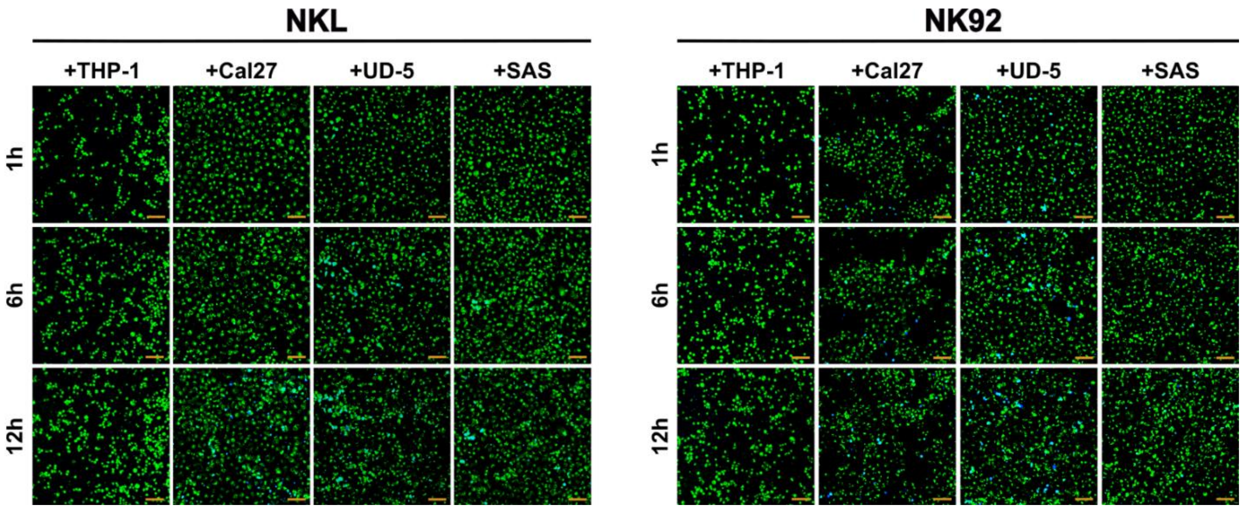

Figure S10. Representative time-lapse fluorescence microscopy to assess NK cell-mediated cytotoxicity against target cancer cells. Unt NKL (left) and NK92 (right) cells were co-incubated with HNSCC target cells (Cal27, UD-5, SAS) at an E:T ratio of 1:1. The THP-1 cells served as control cells. Target cells were pre-labeled with PKH67 (green), and SYTOX™ Blue was added to detect dead cells (blue), thereby enabling visualization of NK cell-mediated cytotoxic effects over time. Monitoring was performed for 20 h, and images captured at 1, 6, and 12 h after co-incubation are shown representatively. The labelled NK cells were excluded from the images to allow better visualization of the dynamic changes in the cancer cells. The scale bar is 100 µm.

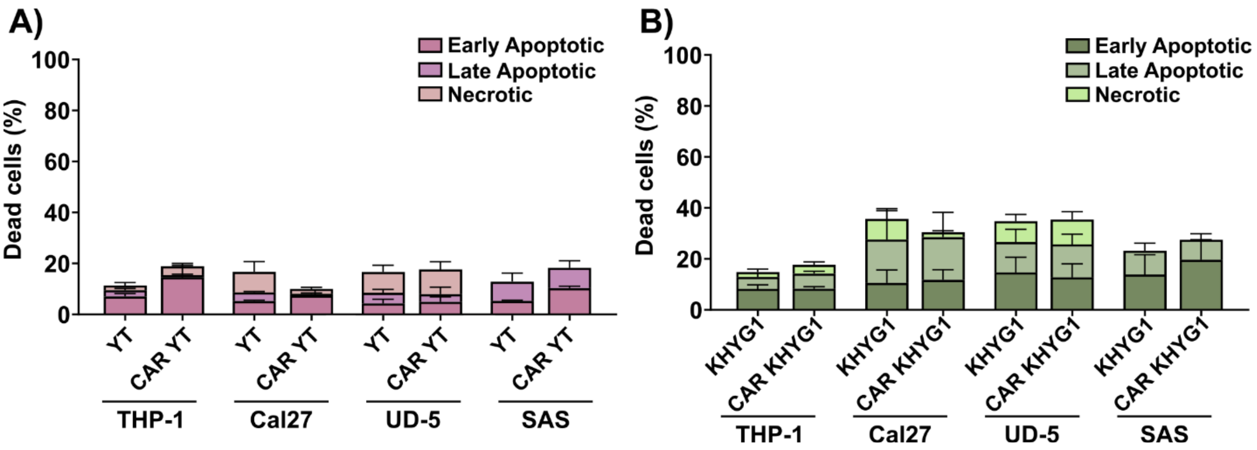

Figure S11. Flow cytometric analysis of apoptotic and necrotic cells stained with Annexin V-FITC and PI. Cal27, UD-5, and SAS target cells (2×10^5 cells per well) with high Hsp70 expression and THP-1 cells with low or no mHsp70 expression were co-cultured with **A)** YT and **B)** KHYG1 (either CAR-transduced or Unt cells) effector NK cells at 37 °C for 6 h at an effector-to-target (E:T) ratio of 1:1 before Annexin V/PI staining and flow cytometry analysis (mean ± SD, n ≥ 3 independent experiments).

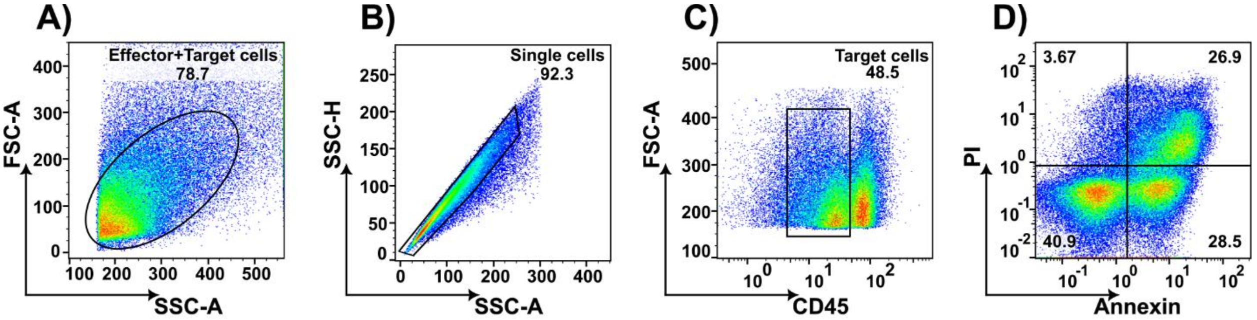

Figure S12. Representative example of gating strategy for flow cytometry analysis of apoptosis and necrosis in target cell lines using an Annexin V/PI assay after a 6-h co-incubation with NK effector cells. A) Effector and target cells were gated based on side/forward scatter properties, **B)** single-cell characteristics, **C)** the leukocyte marker CD45, and **D)** Annexin V-FITC vs PI. Early and late apoptosis, as well as necrosis in the target cancer cell population ($CD45^-$) were identified based on the following staining patterns: viable cells (Annexin $V^–$/ $PI^–$), early apoptosis (Annexin V^+/ $PI^–$), late apoptosis (Annexin V^+/ PI^+), and necrosis (Annexin $V^–$/ PI^+).

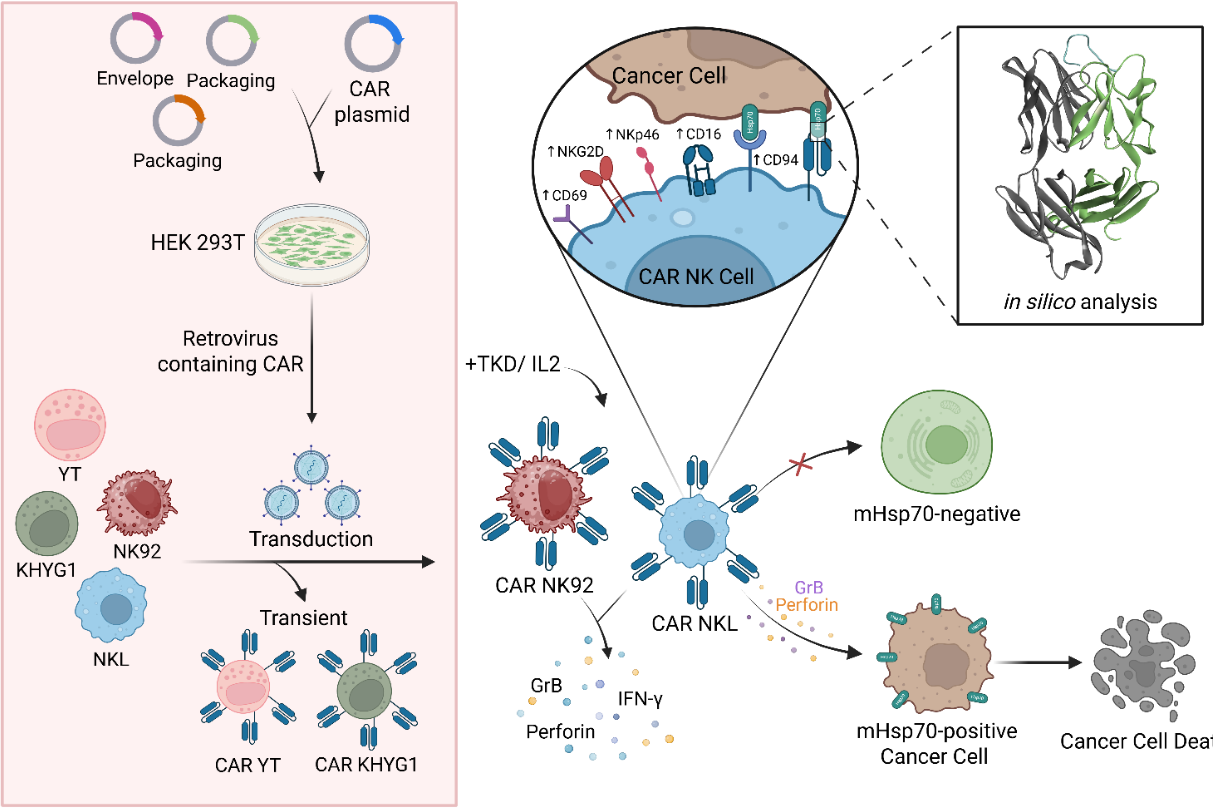

Figure S13. Schematic representation of the experimental workflow used for the generation and functional assessment of anti-Hsp70 CAR engineered NK cells. A second-generation CAR construct targeting mHsp70 was packaged into retroviral particles using HEK 293T cells and transduced into NK cell lines (YT, KHYG1, NKL, NK92). The transduced CAR NK cells were expanded and stimulated with Hsp70-derived TKD peptide and low-dose IL-2, after which their cytotoxic responses against mHsp70-positive HNSCC cell lines were assessed. Functional assays included measurement of effector molecules (e.g., IFN-γ, granzyme B, perforin), Annexin V/PI-based cytotoxicity analysis, and evaluation of antigen specificity using mHsp70-negative cells. Structural insights into CAR-antigen interactions were further supported by molecular docking and molecular dynamics simulation analyses. This figure was created with BioRender.com.

References

1. Bashiri Dezfouli A, Yazdi M, Benmebarek MR, et al. CAR T Cells Targeting Membrane-Bound Hsp70 on Tumor Cells Mimic Hsp70-Primed NK Cells. *Front Immunol*. 2022;13:883694.
2. Rodarte JV, Baehr C, Hicks D, et al. Structures of drug-specific monoclonal antibodies bound to opioids and nicotine reveal a common mode of binding. *Structure*. 2023;31(1):20-32.e5.
3. Pietri GP, Tontini M, Brogioni B, et al. Elucidating the Structural and Minimal Protective Epitope of the Serogroup X Meningococcal Capsular Polysaccharide. *Front Mol Biosci*. 2021;8:745360.
4. Tubiana T, Carvaillo JC, Boulard Y, Bressanelli S. TTClust: A Versatile Molecular Simulation Trajectory Clustering Program with Graphical Summaries. *J Chem Inf Model*. 2018;58(11):2178-2182.
5. Yan Y, Tao H, He J, Huang SY. The HDOCK server for integrated protein-protein docking. *Nat Protoc*. 2020;15(5):1829-1852.
6. Abraham MJ, Murtola T, Schulz R, et al. GROMACS: High performance molecular simulations through multi-level parallelism from laptops to supercomputers. *SoftwareX*. 2015;1:19-25.
7. Lee J, Hitzenberger M, Rieger M, Kern NR, Zacharias M, Im W. CHARMM-GUI supports the Amber force fields. *J Chem Phys*. 2020;153(3):035103.
8. Tian C, Kasavajhala K, Belfon KAA, et al. ff19SB: Amino-Acid-Specific Protein Backbone Parameters Trained against Quantum Mechanics Energy Surfaces in Solution. *J Chem Theory Comput*. 2020;16(1):528-552.
9. Jorgensen WL, Chandrasekhar J, Madura JD, Impey RW, Klein ML. Comparison of simple potential functions for simulating liquid water. *J Chem Phys*. 1983;79(2):926-935.
10. Jo S, Kim T, Iyer VG, Im W. CHARMM-GUI: a web-based graphical user interface for CHARMM. *J Comput Chem*. 2008;29(11):1859-65.
11. Darden T, York D, Pedersen L. Particle mesh Ewald: An N log (N) method for Ewald sums in large systems. *J Chem Phys*. 1993;98:10089-10092.
12. Essmann U, Perera L, Berkowitz ML, Darden T, Lee H, Pedersen LG. A smooth particle mesh Ewald method. *J Chem Phys*. 1995;103(19):8577-8593.
13. Hess B. P-LINCS: A Parallel Linear Constraint Solver for Molecular Simulation. *J Chem Theory Comput*. 2008;4(1):116-22.
14. Bussi G, Donadio D, Parrinello M. Canonical sampling through velocity rescaling. *J Chem Phys*. 2007;126(1):014101.
15. Bernetti M, Bussi G. Pressure control using stochastic cell rescaling. *J Chem Phys*. 2020;153(11):114107.
16. Werner C, Stangl S, Salvermoser L, et al. Hsp70 in Liquid Biopsies-A Tumor-Specific Biomarker for Detection and Response Monitoring in Cancer. *Cancers (Basel)*. 2021;13(15):3706.

17. Kobold S, Steffen J, Chaloupka M, et al. Selective bispecific T cell recruiting antibody and antitumor activity of adoptive T cell transfer. *J Natl Cancer Inst*. 2014;107(1):364.
